# Correction: The effects of tryptophan loading on Attention Deficit Hyperactivity Disorder in adults: A remote double blind randomised controlled trial

**DOI:** 10.1371/journal.pone.0327062

**Published:** 2025-06-25

**Authors:** PLOS One

## Notice of Republication

The title was printed incorrectly. The correct title is: The effects of tryptophan loading on Attention Deficit Hyperactivity Disorder in adults: A remote double blind randomised controlled trial”.

## Supporting information

S1 File Originally published, uncorrected article. (PDF)

S2 File Republished, corrected article. (PDF)
