# Supplementary material for: The effects of tryptophan loading on Attention Deficit Hyperactivity Disorder in adults: A remote double blind randomised controlled trial
Source: PLoS One. 2023 Nov 30;18(11):e0294911. doi: 10.1371/journal.pone.0294911 (PMC10688902; doi:10.1371/journal.pone.0294911)
Supplement: S1 Table — Note that many participants were excluded on multiple grounds. For ease we made exclusions based on the reasons listed in the order listed in the table below. Percentages reflect the percentage of the total number excluded for a group i.e., Control N = 130, ADHD Medicated (N = 227) and ADHD Unmedicated N = 100, as per Fig 1. All percentages are given to the nearest whole number and therefore do not always sum to 100. (DOCX) [file pone.0294911.s001.docx]

S1 Table: Details of reasons for participant exclusion. Note that many participants were excluded on multiple grounds. For ease we made exclusions based on the reasons listed in the order listed in the table below. Percentages reflect the percentage of the total number excluded for a group i.e., Control N = 130, ADHD Medicated (N = 227) and ADHD Unmedicated N = 100, as per Fig. 1. All percentages are given to the nearest whole number and therefore do not always sum to 100.

|  | Exclusions/N (%) | | | |  |
| --- | --- | --- | --- | --- | --- |
|  | | Controls | ADHD Medicated | ADHD Unmedicated | |
| Dietary Intolerances | | 14 (11) | 32 (14) | 2 (2) | |
| Other health conditions | | 31 (24) | 50 (22) | 40 (40) | |
| Other medication | | 7 (5) | 25 (11) | 14 (14) | |
| Smoker | | 8 (6) | 14 (6) | 12 (12) | |
| Following Restricted Diet | | 6 (5) | 0 (0) | 6 (6) | |
| Outside of age range | | 7 (5) | 10 (4) | 5 (5) | |
| ASRS-A outside of range | | 57 (44) | 16 (7) | 11 (11) | |
| Inappropriate ADHD medication use* | | N/A | 43 (19) | 10 (10) | |
| Low ADHD medication adherence | | N/A | 37 (16) | N/A | |

* For the unmedicated group this was use of medication for ADHD within the last three months and for the medicated group, this was medication use that had not been stable for at least one month.
