# Supplementary material for: The effects of tryptophan loading on Attention Deficit Hyperactivity Disorder in adults: A remote double blind randomised controlled trial
Source: PLoS One. 2023 Nov 30;18(11):e0294911. doi: 10.1371/journal.pone.0294911 (PMC10688902; doi:10.1371/journal.pone.0294911)
Supplement: S1 Data — (PDF) [file pone.0294911.s002.pdf]

Full Application Form

Filter Questions

1 Is your study considered research as defined in the guidance icon information?

(•) Yes ( ) No

2 Does your study require external ethical review by either the Health Research Authority (which includes the NHS REC and Social Care REC) or the Ministry of Defence REC?
See guidance icon for further information on the HRA and MOD REC ethical review remit.

( ) Yes
(•) No

Data Collection

3 Select one category from the list below (categories are defined in the guidance icon).

My study involves:

(•) a) Only primary data collection involving human subjects.
( ) b) Only analysis of pre-existing human subject data which is not in the public domain and contains identifiable personal data (see guidance icon for definition)
( ) c) Both primary data collection involving human subjects and analysis of pre-existing human subject data which is not in the public domain and contains identifiable personal data (see guidance icon for definition)
( ) d) Data collection not involving any of the above but presenting sensitive issues
( ) e) None of the above

4 Risk Checklist: Please indicate if your study involves any of the following risks:

- [ ] a) The research involve participants who are vulnerable or unable to give informed consent or in a dependent position.
- [ ] b) Participants will take part in the study without their consent or knowledge at the time of participation or deception of some kind will be involved.
- [ ] c) There is a risk that the research topic might lead to disclosures from the participant concerning their involvement in illegal activities or other activities that represent a threat to themselves or others.
- [ ] d) The study may induce psychological stress or anxiety, or produce humiliation or cause harm or negative consequences beyond the risks encountered in a participant's usual everyday life.
- [ ] e) There is a foreseeable likelihood that a participant's capacity to give fully informed consent may diminish throughout the course of the project i.e. early stage dementia, brain injury etc.
- [ ] f) The study involves imaging techniques such as MRI scans or ultrasound.
- [ ] g) The study involves sources of non-ionising radiation (e.g. lasers)
- [x] h) The study involve physically invasive procedures or the collection of bodily materials (including collection of human tissue for purposes such as DNA/RNA analysis)
- [ ] None of the above.

5 Does the study involve the recruitment of participants under the age of 16?

- ○ Yes
- ● No

Based on your answers to the above filter questions your research has been categorised as High Risk

You can now access an overview of the available sections of the application by selecting the navigate tile in the action panel on the left. Alternatively you can proceed through each section of the application by selecting the next tile.

Upon submission will be subject to review at the next relevant Research Ethics Subcommittee meeting. Meeting dates and submission deadlines can be found here

Section A: General Information

A Applicant Details

Title	First Name	Surname
Dr	Eleanor	Dommett

Department: Department of Psychology

KCL Email: eleanor.dommett@kcl.ac.uk

A2 Applicant Status

Staff

A3 Applicant Role

Principal Investigator

A7 Faculty/Institute/School
Please refer to the information icon if you are unsure of your Faculty/Institute/School.

Psychiatry, Psychology & Neuroscience

A9 Job Title

Reader in Neuroscience

A13

Title	First Name	Surname
Miss	Larisa	Dinu

Organisation: King's College London

Email: larisa.dinu@kcl.ac.uk

What is the role of this investigator?

Research Assistant

Section B: Project Information

B1 Project Title
A working title that accurately reflect the aims of the project.

Effects of acute tryptophan loading and depletion on attention and impulsivity in ADHD

B2 Anticipated start date for the collection of data: 01/05/2020

B3 Expected completion date of the project: 30/04/2023

B4 Is this a funded project?

- ○ Yes
- ◉ No

B5 What are the aims and objectives of the project?

Provide the academic/scientific justification of the project as well as detailing and explaining the principal research question, objectives and hypotheses to be tested.
Please Note: Applications to the BDM and PNM RESC should include a full list of references/citations to back up the academic/scientific justification of the project.

ADHD is a neurodevelopmental disorder characterized by poor attention, heightened impulsivity and hyperactivity (American Psychiatric Association, 2000). Within this, impulsivity can be divided into motor, temporal and cognitive impulsivity (Evenden, 1999; Jentsch et al., 2014; Yang, Vollm, & Khalifa, 2018). Although often considered a childhood disorder, its prevalence in adults is estimated at 2.5-5.2% and, critically, many people who meet the criteria for ADHD in adulthood did not meet the criteria as children (Moffitt et al., 2015). This means that research findings from children with ADHD may not apply to adults with the condition. Irrespective of age, psychostimulants (e.g. amphetamine) are the most common and efficacious treatment. They mainly act on dopamine (DA) and noradrenaline (NA) systems and are effective in over 80% of patients (Dittmann et al., 2014). However, whilst psychostimulants reduce ADHD symptoms, they have side effects ranging from insomnia to tachycardia (Mariani, Mariani, & Levin, 2007) and there are concerns about the drugs being abused (Darredeau, Barrett, Jardin, & Pihl, 2007). It is therefore important that other treatments are explored.

Preclinical studies suggest that other neurotransmitter systems might be involved in ADHD aetiology (e.g. Bolanos et al., 2008) and reduced brain serotonin (5-HT) levels have been reported in a well-validated animal model of ADHD (Sagvolden et al., 2005). Furthermore, altered genes relating to serotonin are implicated in ADHD in human research (Baehne et al., 2009; Grünblatt et al., 2012). Interestingly, dietary precursors can impact the synthesis rate and function of serotonin. Tryptophan (TRP) is such a dietary precursor and an essential amino acid involved in the synthesis of 5-HT which can modulate cognition and behaviour (Silber & Schmitt, 2010). Importantly, for this study, depletion of TRP has been associated with ADHD-like symptoms (Banerjee & Nandagopal, 2015) meaning that increasing tryptophan could offer a novel standalone or adjunct treatment in ADHD.

To date, only a few studies have investigated TRP modulation in adults with ADHD. These studies have largely focused on secondary impairments rather than core symptoms, for example, examining reactive aggression (Kotting et al., 2013; Zimmermann et al., 2012), language processing (Grabemann et al., 2013) and memory processes (Zepf et al., 2013). Only one study to date examine attentional processes (Mette et al., 2013), meaning there is little or no research on the main cognitive symptoms of ADHD. Moreover, in all cases, the studies have used tryptophan depletion to infer a role for tryptophan in treatment, which assumes that those with ADHD have a high enough level of serotonin production for the depletion to offer a significant reduction. This cannot be assumed given that the individuals may have very low levels to begin with. Based on this, the overall aim of the current study is to investigate the effects of moderating tryptophan levels on attention, motor, temporal and cognitive impulsivity in healthy and ADHD participants. To achieve this, we will assess the impact of acute tryptophan loading (low and high TRP supplementation), compared to a balanced (unmoderated tryptophan) control condition on key cognitive ADHD symptoms in:

1. Healthy control participants (HC) and unmedicated adults with a diagnosis of ADHD (ADHD-) to establish the effects of tryptophan on ADHD symptoms.

2. Medicated adults with ADHD (ADHD+) in comparison to unmedicated adults with ADHD (ADHD-) to establish the effectiveness of tryptophan as an adjunct treatment for ADHD.

Specifically, we hypothesise that:

1. Participants with ADHD will perform significantly worse on tasks measuring attention and impulsivity than HC, with ADHD+ performing at an intermediate level between ADHD- and HC.

This hypothesis relates to the fact that ADHD participants will show impaired performance on the tasks used because of their diagnosis and that this will be partially ameliorated by drug treatment. The tasks have been selected because they are known to reveal such impairments and therefore this hypothesis will confirm previous results and allow us to establish baseline performance for the main study hypotheses.

2. There will be a significant effect on attention and impulsivity measures following moderation of TRP levels in HC such that the control condition will produce poorer performance, while the low and high TRP conditions will improve performance in a dose-

dependent way.

Given that we cannot assume typical levels of 5HT production in ADHD, we will first examine the effects of different TRP loads in HC participants to be sure that performance can be moderated in a dose-dependent way initially.

3. There will be a significant effect of TRP moderation on attention and impulsivity measures in unmedicated ADHD, with the high TRP condition significantly improving performance.

This hypothesis relates to the fact that TRP may be a suitable standalone treatment but also that there may be differences in the high TRP condition and the low TRP condition to HC due to different basal levels of 5HT.

4. There will be a significant difference in the effects of TRP moderation on attention and impulsivity measures in medicated and unmedicated adults with ADHD.

This hypothesis relates to the fact that TRP may offer an adjunct treatment by further normalising the behaviours in individuals on medication for ADHD.

References:

American Psychiatric Association. (2000). Attention-deficit and disruptive behavior disorders. In: Diagnostic and statistical manual of mental disorders. 4th ed, text revision. Washington DC, p. 85–93.

Baehne, C. G., Ehlis, A. C., Plichta, M. M., Conzelmann, A., Pauli, P., Jacob, C., ... & Fallgatter, A. J. (2009). Tph2 gene variants modulate response control processes in adult ADHD patients and healthy individuals. Molecular Psychiatry, 14(11), 1032-1039.

Banerjee, E., & Nandagopal, K. (2015). Does serotonin deficit mediate susceptibility to ADHD?. Neurochemistry international, 82, 52-68.

Bolaños, C.A., Willey, M.D., Maffeo, M.L., Powers, K.D., Kinka, D.W., Grausam, K.B. and Henderson, R.P., 2008. Antidepressant treatment can normalize adult behavioral deficits induced by early-life exposure to methylphenidate. Biological psychiatry, 63(3), pp.309-316.

Darredeau, C., Barrett, S. P., Jardin, B., & Pihl, R. O. (2007). Patterns and predictors of medication compliance, diversion, and misuse in adult prescribed methylphenidate users. Human Psychopharmacology: Clinical and Experimental, 22(8), 529-536.

Dittmann, R. W., Cardo, E., Nagy, P., Anderson, C. S., Adeyi, B., Caballero, B., . . . Coghill, D. R. (2014). Treatment response and remission in a double-blind, randomized, head-to-head study of lisdexamfetamine dimesylate and atomoxetine in children and adolescents with attention-deficit hyperactivity disorder. CNS drugs, 28(11), 1059-1069.

Evenden, J. L. (1999). Varieties of impulsivity. Psychopharmacology (Berl), 146(4), 348-361.

Grabemann, M., Mette, C., Zimmermann, M., Heinrich, V., Uekermann, J., Wiltfang, J., ... & Kis, B. (2013). No clear effects of acute tryptophan depletion on processing affective prosody in male adults with ADHD. Acta psychiatrica scandinavica, 128(2), 142-148.

Grünblatt, E., Geißler, J., Jacob, C. P., Renner, T., Müller, M., Bartl, J., ... & Gerlach, M. (2012). Pilot study: potential transcription markers for adult attention-deficit hyperactivity disorder in whole blood. ADHD Attention Deficit and Hyperactivity Disorders, 4(2), 77-84.

Kötting, W. F., Bubenzer, S., Helmbold, K., Eisert, A., Gaber, T. J., & Zepf, F. D. (2013). Effects of tryptophan depletion on reactive aggression and aggressive decision-making in young people with ADHD. Acta psychiatrica scandinavica, 128(2), 114-123.

Mariani, J. J., Mariani, J. J., & Levin, F. R. (2007). Treatment strategies for co-occurring ADHD and substance use disorders. American Journal on Addictions, 16(sup1), 45-56.

Mette, C., M. Zimmermann, M. Grabemann, M. Abdel-Hamid, J. Uekermann, C. S. Biskup, J. Wiltfang, F. D. Zepf, and B. Kis. "The impact of acute tryptophan depletion on attentional performance in adult patients with ADHD." Acta psychiatrica scandinavica 128, no. 2 (2013): 124-132.

Moffitt, T. E., Houts, R., Asherson, P., Belsky, D. W., Corcoran, D. L., Hammerle, M., . . . Caspi, A. (2015). Is Adult ADHD a Childhood-Onset Neurodevelopmental Disorder? Evidence From a Four-Decade Longitudinal Cohort Study. Am J Psychiatry, appiajp201514101266. doi:10.1176/appi.ajp.2015.14101266

Sagvolden, T., Russell, V. A., Aase, H., Johansen, E. B., & Farshbaf, M. (2005). Rodent models of attention-deficit/hyperactivity disorder. Biological psychiatry, 57(11), 1239-1247.

Silber, B. Y., & Schmitt, J. A. J. (2010). Effects of tryptophan loading on human cognition, mood, and sleep. Neuroscience & Biobehavioral Reviews, 34(3), 387-407.

Zepf, F. D., Landgraf, M., Biskup, C. S., Dahmen, B., Poustka, F., Wöckel, L., & Stadler, C. (2013). No effect of acute tryptophan depletion on verbal declarative memory in young persons with ADHD. Acta psychiatrica scandinavica, 128(2), 133-141.

Zimmermann, M., Grabemann, M., Mette, C., Abdel-Hamid, M., Ueckermann, J., Kraemer, M., ... & Zepf, F. D. (2012). The effects of acute tryptophan depletion on reactive aggression in adults with attention-deficit/hyperactivity disorder (ADHD) and healthy controls. PloS one, 7(3).

B6 Where will the research be conducted? i.e in a facility within the college, in a private organisation, in a public place etc

The research will be conducted in the testing labs within the Psychology Department at Addison House, Guy's Campus, King's College London. In case of remote data collection, participants will complete all tasks remotely, in their own homes, whilst being assisted by a member of the research team via Skype.

B7 If outside of the UK, please state the country/countries in which data collection is expected to occur.

N/A

B8 Selection of methodology from list: (select each that applies)

- [x] Questionnaires
- [ ] Semi-structured interviews
- [ ] Unstructured Interviews
- [ ] Focus Groups
- [ ] Observation
- [ ] Clinical Procedures or Interventions
- [ ] Non-clinical Procedures or Interventions
- [ ] Randomised Controlled Trial
- [ ] Oral history
- [ ] Analysis of pre-existing data from human participants
- [ ] Audio/video recording or photography in a public place
- [ ] Audio/video recording or photography in a private place
- [x] Administration of food substances
- [x] Behavioural/Cognitive Testing
- [ ] Other

If you are using any standardised methods for any of the above selected methodologies, please provide an overview of any standardised documentation to be used. Please provide full names and references where appropriate.
Please note you are not required to submit any standardised forms as supporting documents.

Questionnaire:

Participants interested in completing the study will be directed to an online survey which begins with an information sheet and consent form. Participants will have unlimited time to read the study information and, if they choose, give consent to participate. Those giving consent will be then be required to complete a short screening survey consisting of:

1. Confirm inclusion/exclusion criteria (e.g. not gluten or lactose intolerant).
2. Basic demographic questions (age, gender, ethnicity, handedness and years in education)
2. Dietary questions to indicate if the participants is following any special diets at present.
3. A single question asking about ADHD diagnosis (present/absent) followed by the standardised and well-validated Adult ADHD Self-Report Scale (ASRS) (Kessler, Adler, Ames, Demler, Faraone et al, 2005) which provides an indicator of the level of ADHD-like behaviours in those both with and without the condition.

4. Those identifying as having ADHD will be asked to provide details of any medication prescribed for ADHD (dose, duration of use, type used, adherence).

Finally, participants will be asked to provide an email address to be contacted on to arrange the lab visit.

Behavioural/Cognitive Testing:

Several computerised cognitive tests will be carried out before and after ingesting a low TRP protein drink or a high TRP protein drink (or control). All tests are established and well-validated and we currently run them in our laboratory.

1. Attention will be measured using a version of the Continuous Performance Task (CPT), which has been found to be highly sensitive to the deficits in ADHD, including in adults (Barkley, Murphy, & Kwasnik, 1996; Epstein, Johnson, Varia, & Conners, 2001; Gansler et al., 1998). The CPT which best differentiates those with ADHD from healthy controls is the Test of Variables of Attention or TOVA task (Grane, Endestad, Pinto, & Solbakk, 2014). Briefly, participants are required to press a letter on a keyboard to respond to a target (or 'Go') stimulus whilst inhibiting responses to nontarget ('No-Go') stimulus. There are two phases of the task, from which different conclusions can be made: i) Phase 1 – Go signals are infrequent, presented in just 22.5% of the trials; ii) Phase 2 – Go signals are frequent, presented in 77.5% of the trials. The first phase provides information about inattention and the second about motor impulsivity. This task takes around 15 mins to complete.

2. Temporal impulsivity can be measured with the Delay-Discounting Task (DDT). This task provides a reliable and valid measure of temporal impulsivity that is elevated in those with ADHD (Hurst, Kepley, McCalla, & Livermore, 2011). The task requires participants to make choices between hypothetical rewards now or at a point in the future for several different delays e.g. 1 week, 2 weeks, 1 month, 3 months, 6 months and 1 year. Responses are used to calculate the 'indifference point' for each delay, that is the point at which the participant switches to receive the delayed reward. The task takes around 10 mins.

3. Cognitive impulsivity will be measured using the Iowa Gambling Task (IGT) in which participants are shown 4 decks of cards (labelled A, B, C, and D) and asked to choose 100 times from the decks (Kovács, Richman, Janka, Maraz, & Andó, 2017), with the test taking around 15 mins. Each time participants choose a card they can win or lose virtual money. Decks A and B give large gains and large losses, whereas decks C and D give small gains and small losses. Decks A and B are riskier cards as in the short term they win more but in the long term they create an overall loss. The task takes around 10 mins.

Administration of food substances:

Participants will participate in one of three conditions:
1. Control condition – consumption of whey protein drink with balanced amino acid availability (Lieben et al., 2018). This drink will contain 40g of 100% whey isolate protein, unflavoured, naturally containing between 0-566-0.560g TRP.
2. Low TRP condition – consumption of whey protein drink with an added low dose of TRP to increase availability (Lieben et al., 2018). This drink will contain 40g of 100% whey isolate protein, unflavoured, with an added low dose of tryptophan (1.43g, as Lieben et al., 2018).
3. High TRP condition – consumption of whey protein drink with an added high dose of TRP to increase availability (Lieben et al., 2018). This drink will contain 40g of 100% whey isolate protein, unflavoured, with an added high dose of tryptophan (5.24g, as Lieben et al., 2018).

In all cases, participants will arrive at the laboratory and complete the cognitive tests listed above, before being asked to consume one of the drinks indicated. The participant and researcher conducting the data collection will be blind to the condition. The participant will then wait one hour (Lieben et al., 2018) before repeating the tests. Participants will be asked to remain in the testing area at this time but will have access to power and can therefore choose to read or use other devices.

Additional References:

Barkley, R. A., Murphy, K., & Kwasnik, D. (1996). Psychological adjustment and adaptive impairments in young adults with ADHD. Journal of attention disorders, 1(1), 41-54.

Epstein, J. N., Johnson, D. E., Varia, I. M., & Conners, C. K. (2001). Neuropsychological assessment of response inhibition in adults with ADHD. Journal of Clinical and Experimental Neuropsychology, 23(3), 362-371. doi:DOI 10.1076/jcen.23.3.362.1186.

Gansler, D. A., Fucetola, R., Krengel, M., Stetson, S., Zimering, R., & Makary, C. (1998). Are there cognitive subtypes in adult attention deficit/hyperactivity disorder? The Journal of nervous and mental disease, 186(12), 776-781.

Grane, V. A., Endestad, T., Pinto, A. F., & Solbakk, A. K. (2014). Attentional Control and Subjective Executive Function in Treatment-Naive Adults with Attention Deficit Hyperactivity Disorder. PLoS One, 9(12). doi:ARTN e11522710.1371/journal.pone.0115227

Hurst, R. M., Kepley, H. O., McCalla, M. K., & Livermore, M. K. (2011). Internal consistency and discriminant validity of a delay discounting task with an adult self-reported ADHD sample. Journal of attention disorders, 15(5), 412-422.

Kovács, I., Richman, M., Janka, Z., Maraz, A., & Andó, B. (2017). Decision making measured by the Iowa Gambling Task in alcohol use disorder and gambling disorder: a systematic review and meta-analysis. Drug and alcohol dependence.

Kessler, R. C., Adler, L., Ames, M., Demler, O., Faraone, S. et al. (2005). The World Health Organization Adult ADHD Self-Report Scale (ASRS): a short screening scale for use in the general population. Psychological Medicine, 35, 245-256.

Lieben, C. K., Blokland, A., Deutz, N. E., Jansen, W., Han, G., & Hupperts, R. M. (2018). Intake of tryptophan-enriched whey protein acutely enhances recall of positive loaded words in patients with multiple sclerosis. Clinical Nutrition, 37(1), 321-328.

B9 Provide an explanation in lay language outlining each methodology of the study, as identified in question B8.

This study aims to recruit healthy control participants as well as adults with ADHD. The study will begin with a short online screening questionnaire which will allow us to do two things. Firstly, the survey will allow us to confirm key exclusion criteria e.g. gluten intolerance or following a very restrictive diet (i.e. keto). Secondly, the survey will allow us to match the three groups of participants, for example, for age and handedness and collect information on medication in those reporting to have a diagnosis of ADHD.

Eligible participants will then be contacted to attend a 2-hour lab visit at Addison House, Guy's Campus. On arrival at the lab they will be asked to complete three different cognitive tests on a computer. These tests measure attention and impulsivity and will take the person around 30 mins to complete. After completing the baseline tests, in which we would expect participants with ADHD to perform less well than healthy controls, all participants will be asked to consume a drink which will contain differing amounts of the serotonin precursor tryptophan, an amino acid typically found in our diets. Each participant will have just one drink and this may be high, low, or typical in terms of the amount tryptophan. Once they have consumed the drink, participants will wait 60 mins for the drink to be metabolised before completing the three tests again. In total we expect each participants to be in the lab for around 2 hours.

In case of remote data collection, eligible participants will be required to be available for an online testing session which can be done remotely. Testing should take around 2 hours and will take place via the online testing platform, Gorilla, and will also involve a Skype briefing and debriefing at the beginning and end of the allocated time slot. The researcher will ask the participant to prepare and ingest a drink from a sachet (by adding water), which has been sent to them in advance of their online testing appointment. Full instructions on how the drink should be prepared will be provided.

If the summary of your methodology would be supported by a flowchart please attach this here (an editable flowchart can be found via the link in the guidance icon)

Type	Document Name	File Name	Version Date	Version	Size
Other	Tryptophan study flowchart	Tryptophan study flowchart.docx	01/05/2020	1	67.8 KB

B10 I confirm that the researcher who will be administering all tests and/or procedures is competent in the methods.

- (•) Yes
- ( ) No

Section C(I): Participants

C1 Detail your projected number of participants and provide justification for this sample size.
Please note: For projects involving mixed methods and/or multiple participant groups, you should provide an estimate of the number of participants taking part in each method.

Our research uses a 3 x 3 x 2 factorial design. Factor 1, ‘ADHD Status’, is a between-subjects factor and consists of three distinct participant groups (HC, ADHD+, ADHD-). Factor 2 is also between-subjects with three conditions (low TRP, high TRP and TRP norm’. Factor 3 is a within measures factor and includes the before and after consumption measures. There is no existing data to calculate considering these groups and conditions to sample size. However, similar studies covering components of the current work, indicate small-medium effects sizes, therefore a power analysis assuming this effect size and power of 0.95 was conducted. This suggests that we require a total group size of 144 participants (48 HC, 48 ADHD+, 48 ADHD-). We have planned and costed for this +10% as is typical in human work where some individuals may be excluded (e.g. incomplete data set). This gives a total of 158.

C2a What are the Inclusion Criteria? Where appropriate explain how you will screen your participants. (the selection criteria should be clearly defined for multiple participant groups)

All participants:

- Aged between 18-35 years. This age group has been selected to avoid the risk of age-related cognitive decline (Salthouse, 2009).

This will be made clear in the information sheet and we will ask participants to confirm they meet this criteria and provide their age in the screening survey.

Healthy control participants (HC):
- In addition to the above, these participants should not have a diagnosis of ADHD. Scores on the Adult ADHD Self-Report Scale, which can also be used in healthy individuals, will provide confirmation a measure of ADHD-like behaviours in this group (Kessler et al., 2005). Note that this scale is not a diagnostic tool and therefore, although high scores on such a scale may indicate a higher likelihood of ADHD they would not provide any diagnostic information not known to the participant.

ADHD Unmedicated (ADHD-)
- Existing current diagnosis of the ADHD. The extent of ADHD-like behaviours will be assessed using the Adult ADHD Self-Report Scale as described previously.
- Not receiving ADHD medication and have not received any for a period of at least 3 months.

ADHD Medicated (ADHD+)
- Existing current diagnosis of the ADHD. The extent of ADHD-like behaviours will be assessed using the Adult ADHD Self-Report Scale as described previously.
- Currently receiving medication for ADHD (psychostimulant or non-stimulant). Adherence will be confirmed using the approach taken by Safren et al (2007). They adapted a validated scale for medication adherence in HIV to ADHD and used this quick scale to gain a quantitative measure of adherence. This simple scale asks participants the number of pills they should have taken and the number that they have taken in the last two weeks. They noted that almost 80% of individuals reported adherence levels of 80% or more. We will therefore record adherence and consider excluding individuals with less than 80%. By choosing this level, rather than 100% adherence (which is only found in 44% of adults with ADHD) we ensure we have an ecological valid sample. Additionally, participants will be required to take their medication as normal on the day of testing because part of the investigation is to examine the adjunct effects of exercise. They will be asked to confirm this and state the time they last took their medication prior to testing.

Note that the researchers are not allocating to treatment groups. Participants will be allocated based on their existing treatment and those that receive medication will be doing so under the care of their doctor. It is possible to recruit unmedicated individuals with ADHD because many adults choose not to take the psychostimulant treatment.

Additional References:
Safren, S. A., Duran, P., Yovel, I., Perlman, C. A., & Sprich, S. (2007). Medication adherence in psychopharmacologically treated adults with ADHD. Journal of Attention Disorders, 10(3), 257-260.
Salthouse, T. A. (2009). When does age-related cognitive decline begin?. Neurobiology of aging, 30(4), 507-514.

C2b What are the Exclusion Criteria? Where appropriate explain how you will screen your participants. (the selection criteria should be clearly defined for multiple participant groups)

The exclusion criteria for enrollment in this study are:
- gluten or lactose intolerance, as participants will be asked to ingest a whey protein drink;
- current or past diagnosis of nutritional, psychiatric (excluding ADHD) or neurological illnesses;
- being pregnant/breastfeeding;
- being a smoker (including e-cigarettes);
- currently taking medication known to affect the serotonergic system such as antidepressants;
- learning disabilities;
- following a restrictive diet (e.g. keto), as this might interfere with the experiment.

C3 What are the upper and lower age limits? Provide justification for these where appropriate.

Participants will be aged 18-35. This age group is selected to avoid the risk of age-related cognitive decline (Salthouse, 2009).

C4 How will potential participants be identified and approached?
Please note: If different recruitment methods will be used for participant groups, each group should be separately addressed.

Participants will be recruited through institutional volunteer recruitment channels. The institutional recruitment network at King's is highly effective for recruiting healthy participants. Recruitment posters and leaflets will also be advertised on notice boards on campus. We may additionally advertise via websites for support groups e.g. The Adult ADHD Network were we already have research studies advertised and via social media.

C5 Do you have a current or prior relationship with any potential participants? (This includes professional and/or personal relationships)

- ○ Yes, I do have a current or prior relationships with potential participants.
- ◉ No, I do not have any current or prior relationships with potential participants.

C6 Gatekeeper Permission: Will you require an individual or organisation to grant you permission to approach/ access your intended participants? This includes gatekeepers contacting participants on your behalf

- ○ Yes, I will be using a gatekeeper to access potential participants
- ◉ No, I will not be using a gatekeeper to access potential participants

C7 Please specify any incentives being offered and a justification for their use.

Participants will be given a £20 Amazon voucher as a 'thank you' for their time.

Informed Consent

C8 Will informed consent be sought from all participants? ◉ Yes ○ No

C8a How will informed consent be obtained for each data collection method/participant group? Who will take consent and how will it be recorded?
Note: Justification must be provided if you will not be providing all participants with an information sheet and gaining written consent

Consent will first be sought through the screening survey with an online version of the form, after participants have been given the opportunity to read the participant information sheet at their own pace. If eligible and enrolled in the study, consent will be sought again at their arrival at the testing laboratory typically by the Research Assistant on another member of the research team as required. Consent forms will be stored electronically on a restricted-access, password-protected OneDrive or Sharepoint account which will be encrypted using an end-to-end encryption for cloud data. For any data stored on hard drives, this will be encrypted using Bitlocker or similar software.

C9 How long will participants be given to decide if they wish to participate?
Please provide justification if participants will be given less than 24 hours

Consent will first be sought online, through the online screening survey. Therefore, participants can take as long as they wish to consider their participation in the study.
The laboratory visit will also take place at least 24 hours apart from the time participants have completed the screening survey, so that participants can further consider their full participation in the study. Consent is again sought at their arrival at the testing laboratory or at the beginning of the remote testing session.

C10 Detail the process by which participants may withdraw from the research both during the research and after it has been completed. A final withdrawal date should also be provided, after which participants may no longer withdraw their data from the study.

Participants are free withdraw at any point of during study, without having to give a reason. Withdrawing from the study will not affect them in any way.

Participants will be able to withdraw their data up to a month after completion of testing, after which their anonymised data will have been included in analyses and interim reports. If they choose to withdraw from the study, their information will not be retained.

Section D: High Risk Research

D1h Risk Identified: The study involves physically invasive procedures or the collection of bodily materials (including collection of human tissue for purposes such DNA/RNA analysis)

Please indicate which of the following apply:

- [x] Physically Intrusive Procedures
- [ ] Collection of bodily material (including collection of human tissue for purposes such as DNA/RNA analysis)

D1h(a) Identify any risks involved with participation and describe how each will be mitigated. If you are performing multiple procedures or sample collections, each method should be separately addressed.

Participants will be asked to ingest a protein drink. This drink might contain gluten and lactose, therefore individuals with intolerance to these substances are not eligible to participate. This is made clear in the information sheet and consent form, so that individuals with gluten or lactose intolerance do not participate in this research.

As it is possible that some people are not aware of mild food intolerance and might experience minor discomfort (such as bloating) after ingesting protein drinks, we will also ensure that the protein powder that we use (protein isolate instead of protein concentrate) contains minimal amount of these substances.

D1h(b) Outline your protocol if any adverse/incidental findings are made as a result of participation *(Please note: this protocol must also be clear to participants in both your information sheet and consent form.)*

N/A

D1h(c) If applicable, please outline your protocol should an adverse event occur during or as a direct result of participation. *For*

example, a participant faints during the procedure.

We do not anticipate any adverse events to occur as a direct result of participation. The screening survey has been set to identify individuals who have lactose or gluten intolerance and these individuals will not be eligible to partake. The only possible adverse event might be mild bloating if participants are not aware of a mild intolerance. Participants are made aware of this risk prior to participation, although all measures will have been taken by the research team to minimise this risk through sourcing high quality protein powders only.

In the event that participants experience these effects, they will do so in the laboratory (because of the timing of consumption) or in their own homes (if we must collect data remotely). In both cases, they will be able to stop testing and be provided with water and a space to rest until they feel better. Given the low risk of a reaction and the likely mild reactions, no urgent care will be necessary, however, participants will be followed up within 48 hrs to check on their well being and provided with NHS guidance (links) on food intolerance. If participants have any adverse reaction that concerns them, they will be advised to contact their GP.

D2 If there are any additional risks or burdens to participants that have not been addressed above, please provide further details and explain how these risks will be mitigated:

N/A

D3 What are the potential benefits to the participant?

The data collected will provide valuable information about the effects of tryptophan on attention and impulsivity in healthy people and those with ADHD. It is anticipated that the study will provide useful findings about the viability of tryptophan as a standalone or adjunct treatment in ADHD and, therefore, has the potential to be beneficial to individuals diagnosed with the condition in the future.

There are no direct benefits to participants for being part of this study, although those who attend and complete the testing session will receive a £20 Amazon voucher as a 'thank you'.

D4 Will participants be guaranteed complete anonymity in the final report and any further research output/s? (x) Yes ( ) No

D4a Please explain how you will ensure participants remain completely anonymous in the final report or any other research output/s.

All participants will be assigned a unique ID in order to enable the research team to invite eligible participants to the testing session. This file will be stored securely on a password-protected and encrypted cloud service to which only the immediate research team has access. All experimental data included in analysis will be free of any identifiable information. All data presented in the final report or any other research outputs will be presented as averages and group comparisons and will not make reference to individual participants.

Section E(I): General Data Protection Regulation Requirements

E1 Does the project involve the collection and/or use of personally identifiable information?

Personally identifiable information is data that can be used to identify an individual, either directly or indirectly. This may include names, job titles, photos, video or audio recording, email addresses, usernames, IP addresses, DNA or one or more factors specific to the physical, genetic, mental, economic, cultural, or social identity of that person. See guidance icon for examples of personal data.

Please indicate which of the following applies:

- ○ a) The project involves the collection and/or use of personally identifiable information
- ◉ b) Personally identifiable information will only be obtained in order to contact potential participants. No further identifying information will be collected as part of the study.
- ○ c) No personally identifiable information will be collected and/or used for this project

E1b Please indicate which of the following applies:

- ○ The personal data used for recruitment purposes will not be linked to the anonymous data collected from participants and will not be held for any longer than is necessary for the purposes of recruitment.
- ◉ The identifiable information used for recruitment purposes will be linked to the data collected from the corresponding participant/s (this includes linking participant details to raw data for withdrawal purposes)

E1c Important Notice: General Data Protection Regulation requirement

Projects involving the collection and processing of personal data must be registered with the King's Data Protection Register. Researchers must obtain confirmation of KDPR registration prior to commencing data collection in order to comply with the new General Data Protection Regulation.

◉ I confirm that I will submit a King's Data Protection Registration Form prior to commencing collection

Please note: Once you have gained ethical clearance, the important step is that you submit the KDPR form prior to commencing data collection. Providing you have submitted a KDPR for registration, you do not have to await confirmation of registration before commencing data collection.

◉ I confirm that I understand that it is the responsibility of the researcher to ensure that all research data is securely handled and stored during and after the project in compliance with the General Data Protection Regulation (GDPR) and College guidelines:

KCL Research Data Management Guidelines

KCL guidance on the General Data Protection Regulation

Section E(II) Data Handling, Protection and Storage

E2a Will any data from which participants could be identified be published (this could be direct quotes or biographical data)?	○ Yes ◉ No
E3a Will the data be pseudonymised and the identifiable data stored securely and separately from the research data?	◉ Yes ○ No

E5 Where will research data be stored during and after the study is completed?

All data collected through Qualtrics (the screening survey) and Gorilla (cognitive tests) is automatically encrypted. The downloaded research data will then be stored on a password-protected cloud service, which will be further encrypted using Boxcryptor, an end-to-end encryption for cloud data. For any data stored on hard drives, this will be encrypted using Bitlocker or similar software.

The email addresses will be removed from the screening survey data and replaced with a participant ID before storage for the screening data, so that all subsequent data will be associated with only the ID. Email address will be stored separately (with a coding to ID) to allow us to contact the participant. At the end of the study, the file containing email addresses and associated ID will be deleted.

After the study, fully anonymised data sets will be retained for up to 4 years following completion of the study, according to the General Data Protection Regulation (GDPR, 2018). These anonymised data sets will only be accessible to the research team. Should a suitable repository become available for this data, fully anonymised datasets may also be uploaded to a public repository.

E6a Who will have access to participants' personal data during the project?

The researchers will have access to the participants email address and ID to arrange testing which could be linked back to data during the project, although they will be stored separately with research data anonymised.

E7 How long will research data be stored for after the project is completed?
Guidance on data retention periods can be found in the King's Data Retention Schedule

All data will be securely stored in an anonymised form for four years after study completion, according to the General Data Protection Regulation (GDPR, 2018). Should a suitable online data repository be available, a full set of anonymised data will be placed on the platform to allow future accessibility. Data will be securely stored on a password-protected network computer and on a password-protected OneDrive or Sharepoint account to which only the Principal Investigator and key research team members will have access.

E4 Research Dissemination: How will results be disseminated?

- [x] Internal report (thesis)
- [x] Journals
- [x] Conference
- [ ] Other

E8a Will research data be shared with any third parties? (Including for the purposes of transcribing data)

○ Yes ● No

E9b Will data be archived for further use?

○ Yes ● No

Section G: Human Tissue

G1 Does the study involve the use or collection of bodily materials or tissue from a human being?

○ Yes ● No

G2 Does the study involve DNA or RNA analysis of any kind? ( ) Yes (•) No

G3 Are substances or products to be administered? (•) Yes ( ) No

G3a Are substances to be administered which are classified as medicinal products? ( ) Yes (•) No

G3b Are substances to be administered which are not classified as medicinal products? (•) Yes ( ) No

If yes detail the following: Name of the substance(s), Amounts to be administered, Number of times substance will be administered, How the product will be obtained and/or stored, prepared (if appropriate) and dispensed/distributed and Route of administration

This experiment is a randomised and double-blinded study. The participants will receive one of the following drinks on a single occasion:

1. Control/TRP-norm: protein drink prepared from 40g of unflavoured 100% whey protein isolate mixed with 400ml water. This form of whey protein powder is readily available in supermarkets and health food shops.
2. Low TRP drink: protein drink prepared from 40g of unflavoured 100% whey protein isolate mixed with 1.43g of L-Tryptophan mixed in 400ml water. As with the whey protein powder, tryptophan powder is readily purchased from health food shops as a dietary supplement.
3. High TRP drink: protein drink prepared from 40g of unflavoured 100% whey protein isolate mixed with 5.24g of L-Tryptophan mixed in 400ml water.

The products will be sourced from a health store widely available in the UK and will be stored in a cool and dry cupboard in Addison House, Guy's Campus. If participation occurs face-to-face, the drink will be prepared half an hour in advance of ingestion time in one of the kitchens available in Addison House. If participation occurs remotely, sachets with the already mixed powders will be delivered to the participants' home addresses. Each sachet with come with full instructions on how to store and prepare the drinks ahead of the experimental procedures. In this case, participants will mix their own drinks following detailed instructions from the research team. The research team will also contact each participant via Skype before the ingestion of the drink to make sure that participants are adequately briefed and have a full understanding of what is expected of them during testing.

G4 Does the study involve only moderately invasive/intrusive procedures? ( ) Yes (•) No

G5 Does the study involve other invasive/intrusive procedures? ( ) Yes (•) No

Tissue Sites

G6

Organisation: This study will not involve the collection nor the storage of human tissue/bodily materials in any way.

Address: N/A

City: N/A

County:

Postcode: N/A

Email: N/A

G7 Give details of the investigators experience or training which qualifies them to conduct the required procedures.

The members of the research team are experienced in conducting studies involving experimental procedures. All procedures will be piloted before the commencement of recruitment. Furthermore, all experimental procedures will be accompanied by a full set of instructions which will serve as a guide for each testing session.

G8 Provide details of the human tissue licence, where applicable.

N/A

Section H: Insurance, Risks and Ethical Issues

H1 Does the project involve any of the Risk Assessment criteria outlined in the information icon guidance?

(●) Yes ( ) No

H1a I confirm that I will complete a Risk Assessment Form which will be signed by my Supervisor or Head of Department prior to commencing data collection

(●) Yes ( ) No

Please note: Your department should be able to provide you with a Risk Assessment Form. If they are unable to do so, please contact Health and Safety Services for further advice.

H2 I confirm that I have read the exclusion criteria for the College's Clinical Trials and Research Projects Involving Human Subjects Insurance Policy, detailed in the guidance icon, and that:

(●) a) This project meets the inclusion criteria of the policy

( ) b) This project falls under the exclusion criteria and I have gained approval from the Finance Department, as instructed in the guidance icon

( ) c) This project falls under the exclusion criteria but approval has not been granted by the Finance Department

H3 I confirm that my travel insurance arrangements are as follows:

- ○ a) I will secure College travel insurance (see guidance icon for further details)
- ○ b) I will secure personal travel insurance
- ◉ c) I do not require travel insurance as I will conduct the research in my country of legal residence
- ○ d) I will not secure travel insurance

H4 I confirm that if Disclosure & Barring Service clearance is required for my study, this will be obtained prior to the commencement of data collection.

○ Yes ○ No ◉ N/A

H5 I confirm that the No Fault Compensation Scheme will be offered to all UK based participants.

◉ Yes ○ No ○ N/A

H6 Give the details of any other review body approvals or permissions obtained (including gatekeepers, other Ethics Committees, peer review, R&D permission).

The study proposal has been peer reviewed within the Department of Psychology, King's College London.

H7 Give details of any other ethical issues which have not been addressed elsewhere in the application and explain how you will mitigate these risks.

N/A

Section I: Supporting Documents

I1 Participant Information Sheet

Information Sheet templates can be found under 'Recruitment documents'.

Type	Document Name	File Name	Version Date	Version	Size
Participant Information Sheet	Information Sheet V2	Information Sheet V2.docx	01/05/2020	2	69.6 KB

Consent form (if applicable)

I2 Consent form (if applicable)

Type	Document Name	File Name	Version Date	Version	Size
Consent Form	Consent Form V2	Consent Form V2.docx	01/05/2020	2	62.9 KB

Questionnaire/Survey template/s

I4 Questionnaire/Survey template/s

Type	Document Name	File Name	Version Date	Version	Size
Questionnaires	ASRS	ASRS.pdf	13/03/2020	N/A	19.5 KB
Questionnaires	Qualtrics survey V2	Qualtrics survey V2.docx	01/05/2020	2	24.9 KB

Evidence of any other approvals or permissions (includes gatekeeper, R&D, other ethical approvals) (if applicable)

I6 Evidence of any other approvals or permissions (includes gatekeeper, R&D, other ethical approvals)

Approach letters to gatekeeper organisations (if applicable)

I7 Approach letters to gatekeeper organisations

Advertisement document (email, poster, flyer etc) (if applicable)

I8 Advertisement document (email, poster, flyer etc)

Type	Document Name	File Name	Version Date	Version	Size
Advertisement Document	Research advertisement V1	Research advertisement V1.docx	30/03/2020	1	24.7 KB

Cover Letter (for amendments and modifications) (if applicable)

I9 Cover Letter (for amendments and modifications)

Type	Document Name	File Name	Version Date	Version	Size
Other	Cover Letter	Cover Letter.pdf	01/05/2020	1	178.9 KB

Other (if applicable)

I10 Other

Researcher/Applicant

J1 Researcher/Applicant Signature

I undertake to abide by accepted ethical principles and appropriate code(s) of practice in carrying out this study. The information supplied above is to the best of my knowledge accurate. I have read the Application Guidelines and clearly understand my obligations and the rights of participants, particularly as regards obtaining valid consent. I understand that I must not commence research with human participants until I have received full approval from the ethics committee.

Please note that in order to authorise your application you must sign off using your KCL email address i.e. joe.bloggs@kcl.ac.uk and your KCL password.

Signed: This form was signed by Larisa Dinu (larisa.dinu@kcl.ac.uk) on 02/05/2020 5:55 PM
