# Supplementary material for: The effects of tryptophan loading on Attention Deficit Hyperactivity Disorder in adults: A remote double blind randomised controlled trial
Source: PLoS One. 2023 Nov 30;18(11):e0294911. doi: 10.1371/journal.pone.0294911 (PMC10688902; doi:10.1371/journal.pone.0294911)
Supplement: S2 Data — (PDF) [file pone.0294911.s003.pdf]

Research Ethics
Office

Franklin Wilkins Building
5.9 Waterloo Bridge Wing
Waterloo Road
London SE1 9NH
Telephone 020 7848 4020/4070/4077
rec@kcl.ac.uk

KING'S College LONDON

03/05/2023

Dear Eleanor

Reference Number: MOD-22/23-17983

Study Title: Effects of acute tryptophan loading and depletion on attention and impulsivity in ADHD

Modification Review Outcome: Full Approval

Thank you for submitting a modification request for the above study. This is a letter to confirm that your request has now been granted Full Approval. Ethical clearance for the project is now valid until **03/05/2024**.

If you have any questions regarding your application please contact the Research Ethics Office at rec@kcl.ac.uk.

Kind regards

Ms Laura Stackpoole

Research Ethics Facilitator

on behalf of

PNM Research Ethics Subcommittee
