# Supplementary material for: The effects of tryptophan loading on Attention Deficit Hyperactivity Disorder in adults: A remote double blind randomised controlled trial
Source: PLoS One. 2023 Nov 30;18(11):e0294911. doi: 10.1371/journal.pone.0294911 (PMC10688902; doi:10.1371/journal.pone.0294911)
Supplement: S4 Data — (PDF) [file pone.0294911.s005.pdf]

Investigating the effects of acute tryptophan loading on attention and impulsivity in adults with and without Attention Deficit Hyperactivity Disorder (ADHD)

Tryptophan and Attention Deficit Hyperactivity Disorder

Study Protocol: Tryptophan and Attention Deficit Hyperactivity Disorder

FULL STUDY TITLE

Investigating the effects of acute tryptophan loading on attention and impulsivity in adults with and without Attention Deficit Hyperactivity Disorder (ADHD)

SHORT STUDY TITLE

Tryptophan and Attention Deficit Hyperactivity Disorder

Table of Contents

I. FUNDING AND SUPPORT IN KIND 5
II. ROLE OF TRIAL SPONSOR AND FUNDER 5
1 BACKGROUND 5
2 RATIONALE....6
3 OBJECTIVES AND OUTCOME MEASURES....6
3.1 Primary objectives 6
3.2 Secondary objective 6
3.3 Outcome measures 6
3.4 Primary outcomes....6
3.5 Secondary outcome....7
3.6 Table of outcomes 7
4 TRIAL DESIGN....7
5 TRIAL SETTING 7
6 PARTICIPANT ELIGIBILITY CRITERIA....7
6.1 Inclusion criteria....7
6.2 Exclusion criteria....7
7 TRIAL PROCEDURES 8
7.1 Recruitment....8
7.1.1 Participant identification 9
7.1.2 Screening 9
7.1.3 Payment 9
7.2 Consent 9
7.3 Trial assessments....9
7.4 Qualitative assessments....10
7.5 Withdrawal criteria 10
7.6 End of study....10
8 CONCOMITANT MEDICATION 10
8.1 Assessment of medication adherence 10
9 STATISTICS AND DATA ANALYSIS....11
9.1 Sample size calculation 11
9.2 Planned recruitment rate 11
9.3 Statistical analysis plan....11
9.3.1 Primary and secondary outcome analysis....11
10 PARTICIPANT POPULATION....11
11. DATA MANAGEMENT....11
11.1 Data collection tools and source document identification 11
11.2 Data handling and record keeping....11
11.3 Access to Data....12

11.4 Archiving 12
12 ETHICAL AND REGULATORY CONSIDERATIONS 12
12.1 Research Ethics Committee (REC) review& reports 12
12.2 Peer review 12
12.3 Public and Patient Involvement 12
12.4 Data protection and patient confidentiality 12
12.5 Financial and other competing interests 13
12.6 Indemnity 13
12.7 Access to the final study dataset 13
13 DISSEMINIATION POLICY 13
13.1 Dissemination policy 13
13.2 Authorship eligibility guidelines and any intended use of professional writers 13
14 REFERENCES 13

I. FUNDING AND SUPPORT IN KIND

FUNDER(S)<br><br>(Names and contact details of ALL organisations providing funding and/or support in kind for this study)	FINANCIAL AND NON FINANCIALSUPPORT GIVEN
No project specific funding was received.	N/A
King's College London	Facilities for conducting the research study

II. ROLE OF TRIAL SPONSOR AND FUNDER

King's College London is the sponsor of the study, being responsible for conducting the research procedures as described in the protocol, analyses, interpretation, and dissemination of findings. appropriate standards, conduct and reporting are adhered to regarding its facilities and staff involved with the project.

1 BACKGROUND

ADHD is a neurodevelopmental disorder characterized by poor attention, heightened impulsivity, and hyperactivity (American Psychiatric Association, 2000). Within this, impulsivity can be divided into motor, temporal and cognitive impulsivity (Evenden, 1999; Jentsch et al., 2014; Yang, Vollm, & Khalifa, 2018). Although often considered a childhood disorder, its prevalence in adults is estimated at 2.5-5.2% and, critically, many people who meet the criteria for ADHD in adulthood did not meet the criteria as children (Moffitt et al., 2015). This means that research findings from children with ADHD may not apply to adults with the condition. Irrespective of age, psychostimulants (e.g., amphetamine) are the most common and efficacious treatment. They mainly act on dopamine (DA) and noradrenaline (NA) systems and are effective in over 80% of patients (Dittmann et al., 2014). However, whilst psychostimulants reduce ADHD symptoms, they have side effects ranging from insomnia to tachycardia (Mariani, Mariani, & Levin, 2007) and there are concerns about the drugs being abused (Darredeau, Barrett, Jardin, & Pihl, 2007). It is therefore important that other treatments are explored.

Preclinical studies suggest that other neurotransmitter systems might be involved in ADHD aetiology (e.g. Bolanos et al., 2008) and reduced brain serotonin (5-HT) levels have been reported in a well-validated animal model of ADHD (Sagvolden et al., 2005). Furthermore, altered genes relating to serotonin are implicated in ADHD in human research (Baehne et al., 2009; Grünblatt et al., 2012). Interestingly, dietary precursors can impact the synthesis rate and function of serotonin. Tryptophan (TRP) is such a dietary precursor and an essential amino acid involved in the synthesis of 5-HT which can modulate cognition and behaviour (Silber & Schmitt, 2010). Importantly, for this study, depletion of TRP has been associated with ADHD-like symptoms (Banerjee & Nandagopal, 2015) meaning that increasing tryptophan could offer a novel standalone or adjunct treatment in ADHD. To date, only a few studies have investigated TRP modulation in adults with ADHD. These studies have largely focused on secondary impairments rather than core symptoms, for example, examining reactive aggression (Kotting et al., 2013; Zimmermann et al., 2012), language processing (Grabemann et al., 2013) and memory processes (Zepf et al., 2013). Only one study to date examine attentional processes (Mette et al., 2013), meaning there is little or no research on the main cognitive symptoms of ADHD. Moreover, in all cases, the studies have used tryptophan depletion to infer a role for tryptophan in treatment, which assumes that those with ADHD have a high enough level

of serotonin production for the depletion to offer a significant reduction. This cannot be assumed given that the individuals may have very low levels to begin with. Based on this, the overall aim of the current study is to investigate the effects of moderating tryptophan levels on attention, motor, temporal and cognitive impulsivity in healthy and ADHD participants. To achieve this, we will assess the impact of acute tryptophan loading (low and high TRP supplementation), compared to a balanced (unmoderated tryptophan) control condition on key cognitive ADHD symptoms in:

1. Healthy control participants (HC) and unmedicated adults with a diagnosis of ADHD (ADHD-UM) to establish the effects of tryptophan on ADHD symptoms.

2. Medicated adults with ADHD (ADHD-M) in comparison to unmedicated adults with ADHD (ADHD-UM) to establish the effectiveness of tryptophan as an adjunct treatment for ADHD.

Details of supplementation: The balanced condition (BAL) contained only 40g of 100% whey powder which provided balanced amino acid availability and contained 0.566g of TRP. The low loading condition had added TRP resulting in 1.43g of TRP and the high loading condition contained 5.24g of TRP.

2 RATIONALE

Attention Deficit Hyperactivity Disorder (ADHD) affects around 3% of adults and is associated with reduced attention, high levels of impulsivity, hyperactivity and altered reward learning. At present adult ADHD is normally treated with psychostimulant medications (e.g. Ritalin or Adderall) but there are concerns about side effects, abuse potential and they do not work for everyone. It is therefore important to consider alternative approaches. Preliminary research suggests that modulation of tryptophan may be a suitable intervention but there is a lack of controlled research and very little research at all in adults.

3 OBJECTIVES AND OUTCOME MEASURES

3.1 Primary objectives

The purpose of the study is to assess the impact of acute tryptophan on attention and impulsivity in adults. We aim to see if tryptophan loading is effective in reducing the symptoms of ADHD in adults, both alone and in combination with ADHD medication.

3.2 Secondary objective

There are no secondary objectives.

3.3 Outcome measures

- Attention is measured using omission errors and hit reaction time on the Test of Variables of Attention (TOVA). Additionally, d’ prime will be used as a measure of response sensitivity.
- Motor impulsivity is measured using commission errors on the TOVA.
- Temporal impulsivity is measured using the Delay Discounting Task (DDT) and specifically the area under the discounting curve.
- Cognitive impulsivity is measured using the Iowa Gambling Task (IGT) to assess risky decision making using a net score and percentage of risky decisions.

3.4 Primary outcomes

As detailed above.

3.5 Secondary outcome

N/A.

3.6 Table of outcomes

Objectives	Outcome Measures	Timepoint(s) of evaluation of this outcome measure (if applicable)
**Primary Objective:** To assess the impact of tryptophan loading on attention and impulsivity in adults with ADHD, both alone and in combination with ADHD medication (psychostimulants or non-stimulants) with a healthy control (non-ADHD) reference group.	TOVA, DDT, IGT	Tests will be conducted before tryptophan loading at the start of the laboratory session and again 1 hour after consumption of the protein drink.

4 TRIAL DESIGN

Double blind randomised controlled trial with randomisation stratified by group (ADHD-M, ADHD-UM, HC) and gender (male female) into three different tryptophan conditions, creating a 3 (participant group) x 3 (tryptophan modulation) x 2 (time, before and after) design.

5 TRIAL SETTING

This is a single centre research study, taking place in the Department of Psychology, King's College London but the trial is a remote trial and so all testing takes place online (using MS Teams).

6 PARTICIPANT ELIGIBILITY CRITERIA

6.1 Inclusion criteria

The study is recruiting adults aged 18-35 years. Participants will fall into one of the following categories in order to be eligible for the study:

i. No current or previous diagnosis of ADHD.
ii. Current diagnosis of ADHD but not currently receiving any drug treatment for the condition (and have not done so for 3 months).
iii. Current diagnosis of ADHD and currently receiving psychostimulant drug treatment for the condition with at least 70% adherence.

6.2 Exclusion criteria

The exclusion criteria for this study are:

I. gluten or lactose intolerance, as participants will be asked to ingest a whey protein drink
II. current or past diagnosis of nutritional, psychiatric (excluding ADHD) or neurological illnesses
III. being pregnant/breastfeeding
IV. being a smoker (including e-cigarettes)
V. currently taking medication known to affect the serotonergic system such as antidepressants

VI. learning disabilities
VII. following a restrictive diet (e.g., keto), as this might interfere with the experiment

7 TRIAL PROCEDURES

Participants will first complete a brief online screening survey (~10 mins). As part of the survey, participants will answer question to determine eligibility and provide basic demographic information (e.g., age, gender, ethnicity, education level). The final part of the screening survey that all participants will complete includes a question asking them about any current ADHD diagnosis and the Adult ADHD Self-Report Scale, a short survey assessing ADHD symptomology. For those who report a diagnosis of ADHD, details of treatment (e.g. dose, drug) will be required. Finally, participants will be asked to provide an email address so that they can be contacted by the research team to arrange a time for the testing session once their screening survey answers has been reviewed and they have been confirmed as eligible to participate. All those eligible to participate will be invited to attend a 2-hour online testing session. Given the remote nature of the test, a postal address will also be requested to deliver the protein powder from all eligible participants.

On arrival in the online room, the researcher will remind the participant of the study information and consent will be re-confirmed verbally. Participants will also be asked to confirm that they did not consume protein-rich meals, caffeine, or alcohol the day of the session or the previous night. Participants will then be advised to open the protein powder that had been posted to them and mix the powder with 400 ml of water. They are then asked to put this in the fridge until they are instructed to drink it. The participants will then complete the three cognitive tests. After this they will be asked to drink the protein drink within 10 minutes and then wait a further 1 hour before completing the tests again.

Testing will be in two phases - before and after consuming the protein drink – with the same computerised tests completed in both phases and aim to measure attention and impulsivity. Brief descriptions of the tests are given below:

- Test of attention – participants will be asked to press a letter on a keyboard to respond to a target stimulus whilst inhibiting responses to non-target stimulus.
- Tests of impulsivity – two specific tests will be used to measure impulsivity. In the first test participants will be asked to make choices, using keyboard presses, between hypothetical rewards now or at a point in the future for several different delays e.g. 1 week, 2 weeks, 1 month, 3 months, 6 months and 1 year. In the second test participants will be shown 4 decks of cards (labelled A, B, C, and D) and asked to choose 100 times from the decks with two decks giving greater gains and losses. Additionally, the test of attention will also provide a further measure of impulsivity.

7.1 Recruitment

Participants will be recruited through institutional volunteer recruitment channels. The institutional recruitment network at King’s is highly effective for recruiting healthy participants and those with conditions such as ADHD (which is relatively common in the student population). However, we will also advertise the study via groups such as the UK Adult ADHD network (https://www.ukaan.org/otherresearch.htm) which allows research advertisements and has a long-standing link to King’s College and the Institute of Psychiatry, Psychology and Neuroscience. If interested in the study, participants will complete the short

online questionnaire, after which they will be contacted by the researcher who will confirm eligibility. If eligible, the researcher will contact participants to schedule the online testing session.

7.1.1 Participant identification

Potential participants are recruited through (1) advertisements via e-mail circulars, social media platforms and flyers at King's College London, and (2) advertisements via ADHD local/national organisations. The researcher manages all recruitment.

7.1.2 Screening

All participants are asked to fill in a short online screening survey before they are invited for the laboratory visit to ensure that they meet the eligibility criteria.

7.1.3 Payment

Participants who attend and complete the testing session will receive a £20 Amazon voucher as a ‘thank you’ for participating.

7.2 Consent

Informed consent will be taken from all participants twice. As the study involves two parts (an online survey and testing session), written consent is first sought as part of the online survey. Then, consent is re-confirmed verbally at the start of the online testing session. On both occasions, participants are first presented with a written participant information sheet which explains in detail the study and the various tasks the participant will be undertaking (there is a written participant information sheet in the online survey and participants are also given an electronic copy of the information sheet ahead of the test session as part of the booking confirmation). Informed consent is sought by the Research Assistant, who also explains the details of the study to each participant verbally during the online testing session. All participants are encouraged to ask any questions they may have with regards to the study.

Participants can take as long as they need to consider participation in the study, as the information sheet is openly available at the beginning of the online survey, which the participants complete in their own time. At this point, the research team encourages participants to get in touch should they have any questions. If consent is recorded and the participant is eligible to be invited for a lab visit, they will be contacted by the researcher who will schedule the visit at least 5 days apart from the time the online response was recorded (to allow delivery of the protein). Upon arrival in the online room, the Research Assistant checks with each participant if they are still happy to take part in the study.

7.3 Trial assessments

**Intervention or procedure**	**Number of times it is administered**	**Average time taken per procedure**	**Person conducting the procedures and where they take place**
Online questionnaire (includes informed consent)	1	10m	The online questionnaire is hosted on an online survey builder website (i.e., Qualtrics) and can be taken remotely.
Informed consent (again, at lab visit)	1	10m	Verbal consent will be sought by a Research Assistant upon arrival in the online room.

Delay Discounting Task (computer-based)	2	10m	This task is computer-based and performed under monitoring by the Research Assistant during the online testing session. The task is performed before and after protein consumption.
Iowa Gambling Task (computer-based)	2	10m	This task is computer-based and performed under monitoring by the Research Assistant during the online testing session. The task is performed before and after protein consumption.
Test of Variables of Attention (computer-based)	2	22m	This task is computer-based and performed under monitoring by the Research Assistant during the online testing session. The task is performed before and after protein consumption.

Total time to complete the study: approximately 120 minutes including 1 hour break after consumption of the protein drink.

7.4 Qualitative assessments

Not applicable.

7.5 Withdrawal criteria

Participants are free withdraw at any point of the study, without having to give a reason. Withdrawing from the study will not affect participants in any way. Participants can withdraw their data up to three months after completion of testing, after which their anonymised data will have been included in analyses and interim reports. If they choose to withdraw from the study, we will not retain the information they have given thus far.

7.6 End of study

Participants' involvement with the study end immediately after completing the second set of cognitive tests and receiving their reimbursement.

8 CONCOMITANT MEDICATION

The current study is investigating the effects of tryptophan on ADHD core symptoms in medicated and unmedicated individuals with a diagnosis of ADHD. Participants are instructed to take their medication as usual.

8.1 Assessment of medication adherence

While the current study does not involve the administration of any medication, the study is measuring medication adherence of the ADHD medication that some participants are ordinarily taking. This is done in the online screening survey. To measure adherence, participants are asked how many tablets they were supposed to take during the past 2 weeks and how many they took.

9 STATISTICS AND DATA ANALYSIS

9.1 Sample size calculation

There is no existing data to calculate considering these groups and conditions to sample size. However, similar studies covering components of the current work, indicate small-medium effects sizes, therefore a power analysis assuming this effect size and power of 0.95 was conducted. This suggests that we require a total group size of 144 participants (48 HC, 48 ADHD-M, 48 ADHD-UM).

9.2 Planned recruitment rate

Based on availability of researchers to run online testing, we expect to test up to 4 participants per week.

9.3 Statistical analysis plan

9.3.1 Primary and secondary outcome analysis

To achieve the study's objectives, we will adopt a 3 x 3 x 2 factorial design. Factor 1, ‘ADHD Status’, is a between-subjects factor and consists of three distinct participant groups (healthy controls, unmedicated ADHD, medicated ADHD). Factor 2, also between-subjects, is ‘Tryptophan modulation’’ and consists of three conditions: balanced, low loading, high loading. The final factor is within-subjects and is the ‘Time’ at which measures are collected i.e., before and after protein (tryptophan) consumption. Data analysis will use parametric methods, typically mixed ANOVA, after normality checks and any necessary transformations.

10 PARTICIPANT POPULATION

Adults aged 18-35 years with or without a diagnosis of ADHD.

11. DATA MANAGEMENT

11.1 Data collection tools and source document identification

Participant data is collected in the online screening survey via the platform, Qualtrics. This data contains an email address which is only accessible to the research completing screening and allocation and is held securely on university, password-restricted storage. Participant files are produced during each testing session within the online experiment platform (Gorilla). These do not contain identifiable information but rather a participant ID. Documents linking IDs to participants screening data are securely held within a restricted-access OneDrive for Business account which is password-protected and accessible only to the immediate research team (and only to the researcher completing allocation until after all data collection is completed).

11.2 Data handling and record keeping

Participants' anonymised personal data is securely stored on a restricted-access OneDrive for Business folder to which only the immediate research team has access. These files are also stored on password-protected university, personal and laptop computers as necessary for analysis. However, the master spreadsheet which contains the participants' e-mail addresses correlated with their unique IDs is password-protected and only the researcher conducting allocation has access to this.

11.3 Access to Data

Direct access will be granted to authorised representatives from the Sponsor, host institution and the regulatory authorities to permit trial-related monitoring, audits, and inspections- in line with participant consent.

11.4 Archiving

Fully anonymised data will be made available on the institutional repository once the work is published.

12 ETHICAL AND REGULATORY CONSIDERATIONS

12.1 Research Ethics Committee (REC) review& reports

The study has received ethical clearance from the Psychiatry Nursing and Midwifery Research Ethics Subcommittees (PNM RESC) under the reference number HR-19/20-17983.

12.2 Peer review

No external funding was required for this research and ask such no funder peer review occurred. The quality of the research was assessed within the research team, between the Principal Investigator and the co-investigators (at King's College and Sheffield University).

12.3 Public and Patient Involvement

We plan to work with people with lived experiences in undertaking the research and dissemination of findings through existing links to various groups including the Young People’s Mental Health Advisory Group. The members of the group are aged 16-26, which overlaps very well with our target sample (18-35). We are hoping that we can discuss our project in more detail and feedback their advice into how we are conducting the study, how can we make the project more enjoyable for individuals with lived experiences of ADHD and what possibilities of dissemination are optimal for reaching a larger, more diverse audience.

12.4 Data protection and patient confidentiality

Data will be processed in accordance with the General Data Protection Regulation (GDPR, 2018) and the Data Protection Act (2018).

On receipt of screening survey data, email addresses will be removed from the dataset and replaced with a participant ID, meaning screening data will be stored anonymously. This ID will then be used for all data collected. Email addresses will be stored separately to allow participants to be contacted for booking in testing times. During the study all data will be stored on secure university servers, accessible to only the research team.

At the end of the study, email addresses will be deleted, and fully anonymised data will be stored on the university servers for up to five years after publication of the work, accessible only to the researchers. Should a suitable online data repository be available, a full set of anonymised data will be placed on the platform to allow future accessibility (likely institutional repository).

Participants will not be identifiable from any outputs of the project (e.g. report).

The only individuals who will have access to the personal data of the participants are those involved in allocation of the participants to conditions and booking them in.

The data generated by the study will be analysed by the Research Assistant, Principal Investigators and undergraduate and postgraduate students. All datasets will be fully anonymised. All data will be fully

anonymised for analysis. The personal information that will be published e.g., age, sex, ethnicity and years of education, which will represent group averages and will not refer to individual participants.

The researcher will act a custodian for the data generated by the study. Contact details are:

Dr Eleanor Dommett

Depart of Psychology

Institute of Psychiatry, Psychology & Neuroscience

Guy's Campus, 2nd floor Addison House

SE11UL

Eleanor.dommett@kcl.ac.uk

12.5 Financial and other competing interests

There is no conflict of interest.

12.6 Indemnity

The sponsor, King's College London, will take primary responsibility for ensuring that the design of the study meets appropriate standards and that arrangements are in place to ensure appropriate management, design, conduct and reporting. King's College London provides cover under its No Fault Compensation Insurance, which provides for payment of damages or compensation in respect of any claim made by a research subject for bodily injury arising out of participation in human volunteer studies (with certain restrictions).

12.7 Access to the final study dataset

The final trial dataset will be fully anonymised. The Principal Investigator will have access to the final dataset. A copy of the anonymised dataset might be published on an online data repository if a suitable one becomes available.

13 DISSEMINIATION POLICY

13.1 Dissemination policy

The results will be disseminated though internal reports, peer-reviewed journals and at national and international conferences. This research study will lead to the generation of intellectual property, which will be the property of the Principal Investigator.

13.2 Authorship eligibility guidelines and any intended use of professional writers

Authorship will be granted to members of the research group who have made an important contribution to the conduct, analysis and dissemination of the study’s findings.

14 REFERENCES

American Psychiatric Association. (2000). Attention-deficit and disruptive behavior disorders. In: Diagnostic and statistical manual of mental disorders. 4th ed, text revision. Washington DC, p. 85–93.

Baehne, C. G., Ehlis, A. C., Plichta, M. M., Conzelmann, A., Pauli, P., Jacob, C., ... & Fallgatter, A. J. (2009). Tph2 gene variants modulate response control processes in adult ADHD patients and healthy individuals. Molecular Psychiatry, 14(11), 1032-1039.

Banerjee, E., & Nandagopal, K. (2015). Does serotonin deficit mediate susceptibility to ADHD?. Neurochemistry international, 82, 52-68.

Bolaños, C.A., Willey, M.D., Maffeo, M.L., Powers, K.D., Kinka, D.W., Grausam, K.B. and Henderson, R.P., 2008. Antidepressant treatment can normalize adult behavioral deficits induced by early-life exposure to methylphenidate. Biological psychiatry, 63(3), pp.309-316.

Darredeau, C., Barrett, S. P., Jardin, B., & Pihl, R. O. (2007). Patterns and predictors of medication compliance, diversion, and misuse in adult prescribed methylphenidate users. Human Psychopharmacology: Clinical and Experimental, 22(8), 529-536.

Dittmann, R. W., Cardo, E., Nagy, P., Anderson, C. S., Adeyi, B., Caballero, B., . . . Coghill, D. R. (2014). Treatment response and remission in a double-blind, randomized, head-to-head study of lisdexamfetamine dimesylate and atomoxetine in children and adolescents with attention-deficit hyperactivity disorder. CNS drugs, 28(11), 1059-1069.

Evenden, J. L. (1999). Varieties of impulsivity. Psychopharmacology (Berl), 146(4), 348-361.

Grabemann, M., Mette, C., Zimmermann, M., Heinrich, V., Uekermann, J., Wiltfang, J., ... & Kis, B. (2013). No clear effects of acute tryptophan depletion on processing affective prosody in male adults with ADHD. Acta psychiatrica scandinavica, 128(2), 142-148.

Grünblatt, E., Geißler, J., Jacob, C. P., Renner, T., Müller, M., Bartl, J., ... & Gerlach, M. (2012). Pilot study: potential transcription markers for adult attention-deficit hyperactivity disorder in whole blood. ADHD Attention Deficit and Hyperactivity Disorders, 4(2), 77-84.

Kötting, W. F., Bubenzer, S., Helmbold, K., Eisert, A., Gaber, T. J., & Zepf, F. D. (2013). Effects of tryptophan depletion on reactive aggression and aggressive decision-making in young people with ADHD. Acta psychiatrica scandinavica, 128(2), 114-123.

Mariani, J. J., Mariani, J. J., & Levin, F. R. (2007). Treatment strategies for co-occurring ADHD and substance use disorders. American Journal on Addictions, 16(sup1), 45-56.

Mette, C., M. Zimmermann, M. Grabemann, M. Abdel-Hamid, J. Uekermann, C. S. Biskup, J. Wiltfang, F. D. Zepf, and B. Kis. "The impact of acute tryptophan depletion on attentional performance in adult patients with ADHD." Acta psychiatrica scandinavica 128, no.2 (2013): 124-132.

Moffitt, T. E., Houts, R., Asherson, P., Belsky, D. W., Corcoran, D. L., Hammerle, M., . . . Caspi, A. (2015). Is Adult ADHD a Childhood- Onset Neurodevelopmental Disorder? Evidence From a Four-Decade Longitudinal Cohort Study. Am J Psychiatry, appiajp201514101266. doi:10.1176/appi.ajp.2015.14101266

Sagvolden, T., Russell, V. A., Aase, H., Johansen, E. B., & Farshbaf, M. (2005). Rodent models of attention-deficit/hyperactivity disorder. Biological psychiatry, 57(11), 1239-1247.

Silber, B. Y., & Schmitt, J. A. J. (2010). Effects of tryptophan loading on human cognition, mood, and sleep. Neuroscience & Biobehavioral Reviews, 34(3), 387-407.

Zepf, F. D., Landgraf, M., Biskup, C. S., Dahmen, B., Poustka, F., Wöckel, L., & Stadler, C. (2013). No effect of acute tryptophan depletion on verbal declarative memory in young persons with ADHD. Acta psychiatrica scandinavica, 128(2), 133-141.

Zimmermann, M., Grabemann, M., Mette, C., Abdel-Hamid, M., Ueckermann, J., Kraemer, M., ... & Zepf, F. D. (2012). The effects of acute tryptophan depletion on reactive aggression in adults with attention-deficit/hyperactivity disorder (ADHD) and healthy controls. PloS one, 7(3)
