# Supplementary material for: The effects of tryptophan loading on Attention Deficit Hyperactivity Disorder in adults: A remote double blind randomised controlled trial
Source: PLoS One. 2023 Nov 30;18(11):e0294911. doi: 10.1371/journal.pone.0294911 (PMC10688902; doi:10.1371/journal.pone.0294911)
Supplement: Attachment — Submitted filename: Response to Reviewers.docx [file pone.0294911.s006.docx]

**Journal requirement changes:**

When submitting your revision, we need you to address these additional requirements.

1. Please ensure that your manuscript meets PLOS ONE's style requirements, including those for file naming. The PLOS ONE style templates can be found at

and

<https://journals.plos.org/plosone/s/file?id=ba62/PLOSOne_formatting_sample_title_authors_affiliations.pdf>

We believe that we have now formatted this according to the requirements.

2. We note that you have stated that you will provide repository information for your data at acceptance. Should your manuscript be accepted for publication, we will hold it until you provide the relevant accession numbers or DOIs necessary to access your data. If you wish to make changes to your Data Availability statement, please describe these changes in your cover letter and we will update your Data Availability statement to reflect the information you provide.

Please note we have now placed all our data on the Open Science Framework and so it is now freely available and publicly accessible. The doi is 10.17605/OSF.IO/3PY72 and the url for the project page (including data) is <https://osf.io/3py72/>

3. Please include captions for your Supporting Information files at the end of your manuscript, and update any in-text citations to match accordingly. Please see our Supporting Information guidelines for more information: <http://journals.plos.org/plosone/s/supporting-information>.

We have now added one supporting information file in response to the reviewers. The caption for this is at the end of the file as requested. All references have been updated to reflect the changes detailed below.

**Please note that all line numbers refer to the marked up (tracked changes) version.**

**Reviewer 1** (Huey-Ling Chiang):

This is the first study to adopt a double blind randomized controlled trial to examine the effects of acute tryptophan (TRP) loading on attention and impulsivity in adults with ADHD. They found no effects of TRP were observed for any of the measures. The strength of this study is to use validated neuropsychological measures of the inattention and impulsivity, to include individuals with ADHD taking medication and those not taking any, and to utilize clear study protocol and TRP loading paradigm. Although the limitations of the current study are appropriately discussed, I still have some major concerns.

Although the authors stated that the study utilised an existing loading paradigm which has been verified with plasma measurements of TRP previously, the cognitive tasks were conducted 3 hours after dietary intake in a sample of patients with multiple sclerosis in the study mentioned by the author (1). Another study using acute tryptophan loading approach measured pharmacokinetics of the acute tryptophan intervention showing that TRP reached maximum plasma levels of free tryptophan at 3 hours, and connectivity changes of resting‐state MRI was observed 3 hours after the ingestion (2). Additionally, the effect of TRP loading could have been small to the brain, even though plasma TRP was high. However, the authors choose to measure inattention and impulsivity again 1 hour followed by consumption of a protein drink. Could the authors explain why they decide to wait only1 hour before completing the tests again? Is there more evidence supporting the study design?

Ref 1: Lieben CK, Blokland A, Deutz NE, Jansen W, Han G, Hupperts RM. Intake of tryptophan-enriched whey protein acutely enhances recall of positive loaded words in patients with multiple sclerosis. Clin Nutr. 2018 Feb;37(1):321-328.

Ref 2: Deza-Araujo YI, Neukam PT, Marxen M, Müller DK, Henle T, Smolka MN. Acute tryptophan loading decreases functional connectivity between the default mode network and emotion-related brain regions. Hum Brain Mapp. 2019 Apr 15;40(6):1844-1855.

The reviewer is quite correct that the study we reference (Lieben et al., 2018) included cognitive testing completed three hours after intake. It also included other measures collected at 2 and 4 hours. Critically though, the plasma levels of tryptophan were collected at 1, 2, 3 and 4 hours after consumption and these generally peaked at 1 hour before dropping off again by 3 hours. We thank the reviewer for drawing our attention to the work by Deza-Araujo et al. (2019) which we had previously omitted. We notice that they also reviewed blood plasma levels of tryptophan at 1-, 3- and 6-hours post consumption and found a substantial increase in blood plasma levels following their loading paradigm within one hour as well. They completed behavioural assessments at various time points, including after 1 hour. The data for this is not presented, although they do state no findings were significant after accounting for multiple comparisons.

We based our decision to test after 1 hour on the blood plasma levels in the Lieben et al., study and to a lesser degree (given the slight variation in drink make-up) on earlier work by Yuwiler et al. (1981) who demonstrated that after administering a tryptophan drink, blood plasma levels were increased by almost 3 times basal levels by 1 hour after consumption. These findings, combined with animal work suggesting a close correlation between blood and brain levels, informed our choice. The animal work includes Fernstrom and Wurtman (1971) who showed that brain serotonin levels rise within 30 mins of an intra-peritoneal injection of tryptophan in rats. Critically though, they note a good correlation between blood plasma and brain levels of tryptophan with both rising on a comparable timescale after an injection. This is further supported by a similar study by Tagliamonte et al. (1973). Both studies used injections so the absolute times will not generalise to the current study but other work by Fernstrom et al. (1973) used oral administration in rats and again found temporally comparable blood and brain rises. Clearly, translating between species is difficult but putting all this together we felt justified in using the one-hour time point. However, we completely accept that peak plasma may not correspond to peak brain levels and that this is a limitation of the study. As such to address this comment we have done two things. Firstly, we have included a more extensive justification of our design choice including the references noted here [Lines 233-237]. Secondly, we have acknowledged that this is a potential limitation of the work in the discussion [Lines 498-507].

**Reviewer 2** (Mussie Msghina):

The authors conducted a randomized double-blind study to investigate manipulation of plasma tryptophan halt on measures of inattention and impulsivity in healthy participants and medicated and unmedicated ADHD patients between the age of 18-35. To this end, they used three paradigms to measure inattention and impulsivity related outcome variables. The study is generally well conducted in its rigor, and well described in the method part of the manuscript, but a number of concerns and inconsistencies weaken the conclusions.

We are grateful to the reviewer for their comments throughout, which we believe have substantially improved the manuscript.

What is the basic hypothesis of the study? This is not clearly stated.

We have now included an explicit hypothesis at the end of the introduction [Lines 121-123] and remind the reader of this hypothesis at the start of the discussion as well [Lines 375-377].

Are the selected outcome variables, as challenged by the experimental paradigm, serotonin synthesis-dependent? Signal substances are prefabricated and stored in dense-core vesicles, with a large releasable pool of pre-synthesized transmitter. All serotonin dependent functions under all experimental conditions cannot simply be assumed to be synthesis dependent.
The authors thus need to clearly state a hypothesis and discuss (or cite literature, if there is any) whether the outcome variables they chose to measure, as challenged by experimental paradigm they employ, are expected to be dependent on ongoing serotonin synthesis. This seems to be a tacit assumption, but it has to be clearly spelled out in the introduction. For example, studies have shown that cognitive control and stress response under certain conditions are dopamine synthesis dependent. Authors thus need to cite literature establishing some sort of correlation / dependency between serotonin synthesis and the measured outcome variables, or at the very least discuss this in the introduction together with their hypothesis.

In the introduction we have focused on providing a concise background and rationale for the current study. We have drawn on a range of literature to demonstrate the potential importance of serotonin in ADHD and the prior work on TRP and ADHD. We have now added a hypothesis as indicated above. We completely understand the reviewer’s point about whether the tasks we selected are in fact dependent on serotonin synthesis as opposed to, for example, synaptic serotonin levels. We have now added discussion of this in the methods section. We have opted to put it in the methods rather than the introduction because that is where we describe the specific tasks and so felt it most logically fitted. We hope that this is acceptable.

Clearly, whether a specific task is dependent on synthesis rather than just synaptic levels is very difficult to assess in humans. However, we have attempted to draw on human and non-human literature to support our task selection. Specifically, we have:

- Added references to a paper showing that the TOVA task is sensitive to TRP depletion effects which is indicative of it being dependent on serotonin synthesis rather than just synaptic serotonin levels [Lines 246-248].
- Added reference to a study showing discounting function has been shown to be sensitive to serotonin depletion in rats to support use of the DDT [Lines 263-265].
- Added reference to a study showing decision making as required for the IGT has been linked to serotonin synthesis in a clinical population [Lines 275-277].

As the reviewer intimated in their comments there is not extensive evidence of a link to synthesis as opposed to synaptic levels so whilst we have acknowledged the evidence that is available, we have also included evidence that increased synthesis does indeed lead to increased release of 5-HT within the introduction [Lines 90-93]. Finally, we have noted that the limited evidence for the tasks being 5-HT synthesis dependent (as opposed to release/synaptic level dependent) and discussed that even if they increase release this may be counteracted as a limitation of the study [Lines 475-485].

The ASRS scores in medicated and unmedicated ADHD patients are almost exactly the same, which is rather strange with 90% treatment adherence as they report in the results section (line 306). CS medications have high response rate (70-80%), when response is defined as a minimum 30% reduction in symptom scores. With 90% adherence, you would expect to see a statistically significant difference between medicated and unmedicated ADHD patients, otherwise the authors could also conclude from their study that medication (amphetamine and methylphenidate) is ineffective in reducing ADHD symptom scores. Authors need to explain this discrepancy, which may put into question the validity of their study and indicate a flaw in the design.

We thank the reviewer for noting this point. It is something that we have found in several of our studies to date and have therefore considered previously. It is logical to expect that those receiving medication would have lower ASRS scores than those not receiving it. However, that assumes that all individuals will choose to take medication. We think one explanation for this finding is that only those with more severe ADHD choose to take medication and so, whilst the medication lowers symptoms, it may only lower them to a level that is commonly found in those who opt not to take medication, rather than remove them completely. Indeed, most individuals with ADHD will experience residual symptoms even with medication so we would not expect a reduction to sub-clinical levels. Clearly, we cannot be certain that this is the case because our study collected data at one point in time, so we do not have data for the medicated individuals before they took medication. Therefore, to address this comment we have added a section to the discussion [Lines 452-463] where we discuss this lack of difference and explain why it may have arisen but also cite several papers that also report a lack of effect on the ASRS or medication to demonstrate that this is not the only study to report this.

Authors correctly claim that some of the paradigms used in the study are robust enough to differentiate controls from ADHD patients, but this doesn’t seem to be the case in present study as the authors report no differences between the groups even in the pretreatment phase (baseline). If there were any group differences at baseline between healthy controls and ADHD patients, these should be reported as otherwise the paradigms might not have given expected results in the first place, i.e. before TRP modification is even employed. If medication cannot differentiate between treated and untreated patients and robust paradigms cannot differentiate between controls and cases, the value of the finding that TRP had no effect gets weakened in my opinion.

The data are correctly reported in that there were no differences at baseline between the controls and the ADHD groups. This can be deduced from the 2 x 3 x 3 ANOVA we did but we also double checked with One Way ANOVAs on all baseline measures. This was not presented because it duplicates the wider analysis, but we can confirm this here.

We selected the paradigms based on previous research which had shown that the two groups were differentiated but we did not find this to be the case in our hands. Note that we had previously unintentionally omitted the references to support the claim that the TOVA can differentiate between the ADHD and control groups, but we have now added these in [Line 245]. We recognise that the absence of effects may suggest that the paradigms chosen cannot differentiate between the groups. However, that does not mean that they cannot differentiate the within-subjects effects i.e., before and after TRP which is the main focus of the study (aligning with the hypothesis that has now been explicitly stated in the introduction). We have, however, added in a discussion around this point referring to various previous studies and exactly what differences these found [Lines 407-440].

Minor details
- On line 59, authors mention “limited efficacy” of CS medication as a rationale for testing other potential interventions, yet on line 51 mention that 80% respond to CS medication. Authors should either explain how 80% response rate indicates “limited efficacy” or rephrase the statements.

We apologise this was not clear. Whilst 80% of individuals do show a response, most will experience some residual symptoms i.e., the treatment does not remove all symptoms. This combined with side effects and some not being able or willing to take the medication means that other options must be explored. We have re-phrased this to make it clearer, as we agree this was previously misleading [Lines 59-60].

- On line 55-56, authors state that “some patients experience only a 30% symptom reduction, demonstrating treatment needs are not always met”, but that is often the definition criterium for response (30% reduction). This should be rephrased to make it more consistent with the definition of response in the literature.

We do not agree that this is creating inconsistency. A 30% reduction in symptoms may be enough to consider a treatment as working but it is still not fully meeting the treatment needs because significant symptoms remain. However, we appreciate that this phrasing may feel contradictory to some readers and have therefore adjusted the phrasing as requested [Line 55-56].

**Reviewer 3:**

In this manuscript, the authors examine the effect of acute TRP modulation on ADHD symptoms in adults, focusing on attention and impulsivity. They performed a remote double-blind trial with 3 TRP loading doses (balanced, low, and high) in adults with and without ADHD symptoms. Their results showed no effects of TRP loading on the relevant outcome measures of attention and impulsivity in any of the groups.

This is an interesting and timely study, which addresses an important and novel question in the adult ADHD literature. The methodology is generally sound, and the results are clearly described. There are however a few limitations that somewhat reduce my enthusiasm from the manuscript, which should be addressed in a revision. Specifically:

1. Introduction and rationale for the study:

The introduction nicely outlines the rationale for the potential involvement of 5-HT in ADHD and why the concentration of TRP can be relevant. However, from the introduction it is not clear that a one-time consumption of TRP may have any effect, and that it is more about a long-term diet. The authors should better explain why they hypothesize that a one-time consumption may have an effect on behavioral tests as the ones used here. In the 2017 paper cited for the interval used (ref [52]) it is mentioned that executive functions did not change in this interval – why did the authors hypothesize that they would? Also the design does not allow us to know whether participants with ADHD had lower TRP levels to begin with. If not – why would one assume that TRP loading would be beneficial?

We think that we have addressed this to some degree in response to Reviewer 2 where we added quite extensive detail into the methods section explaining why we thought the tasks used would be sensitive serotonin synthesis and why we selected the time interval that we did. This information is covered in Lines 246-248 (TOVA), Lines 263-265 (DDT), Lines 275-277 (IGT) and Lines 233-237 (time interval). We have also adjusted the introduction to emphasize that previous work had found acute changes in TRP or 5-HT had impacted measures hence we opted to use an acute paradigm [Lines 79-81, 119-120]. We had already covered the limitation that the acute treatment may be insufficient in the discussion [Lines 508-517]. We have now added into the discussion the limitation that we did not establish TRP levels at baseline and so cannot confirm that levels are lower in the ADHD sample used in our study, as has been found in the previous work (which we had referenced in the introduction previously – see Lines 94-98).

2. Methods:

- The clinical trial was pre-registered, and no changes were made to the registered protocol, which is good. Still, there are some weaknesses in the methodology, mainly stemming from the remote application of the trial. For example, the authors needed to rely on self-report regarding protein consumption the night before and did not have any physiological measures to examine baseline or post-treatment differences. These are significant weaknesses in the methodology that should be acknowledged and reduce from the potential impact of the outcome.

We recognise that remote data collection and lack of physiological measures is a limitation. We have already discussed the fact that remote data collection can be both a strength and limitation of the design, with the limitation part on Lines 558-567], however, we have now added in the additional limitation of the lack of physiological measures of plasma TRP (we had already discussed the need to look at dietary information and TRP2 genotyping elsewhere) [Lines 567-578].

- The description of what does the 3 X 3 X 2 design mean should come earlier in the text – only in the statistical analysis section it becomes clear, but should be made clearer before (e.g., on p.5).

We have moved this explanation to the Trial Design section [Lines 127-131] and adjusted the original text in the statistical analysis section to align with this.

- Power analysis – I am a bit unclear about how the power was derived. Which calculator was used for it? And for the effect sizes used – which comparisons were taken, given the multiple groups here?

We apologise for not including the specific calculator used. We have now added this – it was G*Power. We used an effect size from the work by Mette et al. (2013), specifically their changes in the ADHD group following acute TRP depletion (as no loading studies had been done). As specified in the manuscript we powered for interaction effects to detect the interaction between group, TRP load and time. Given our hypothesis was that we would see a change in attention and impulsivity in a dose dependent manner with TRP this was the required comparison (i.e., to detect an interaction between TRP and Time). Note that in response to Reviewer 2, this hypothesis is now explicitly stated which hopefully also helps address this point. The same parameters for effect size and power would have allowed detection of within-subjects effects with a sample N = 63 so our selected interaction effect sample size should also have been sufficient for this. However, we accept that we may have needed a larger sample to detect the between factors differences (N = 288). We have now explained this in Lines 155-158 and explained the limitation for between factors in the section of the discussion where we had previously discussed power [Lines 553-557].

- Inclusion criteria – the participants did not go through SCID to confirm current ADHD diagnosis – this is a limitation that should be noted.

This is correct, we asked participants with ADHD to confirm they had received a formal clinical diagnosis previously and then used the ASRS to confirm this. The lack of SCID is indeed a limitation. We had previously noted that our lack of diagnosis as part of the study was a limitation, but we have now explicitly made reference to the need for SCID [Lines 583-584].

Also, excluding participants over 35 due to age-related cognitive decline seems a bit excessive – please reconsider phrasing.

We are not quite sure what the reviewer would like us to rephrase here because this was the reason for the age criteria and we have included a reference to support the onset of age-related decline at this age. Given we cannot change the criteria and we have evidenced the research behind it, we have tried to soften the language around it [Line 170]. We did wonder if the reviewer intended us to mention that this age range was a limitation, so we have now included that in the discussion [Lines 579-581]. If we have misunderstood what was required here, we are happy to make further amendments.

- When gender’is mentioned – do the authors refer to ‘sex’?

The survey question referred to gender i.e. we asked participants to indicate their gender with male or female as answer options. We recognise that we could have offered more options and had a separate question about sex and would do this differently in future but for now we have just had to make the fact we asked about gender clearer in the manuscript [Lines 193-195].

- The outcome measures are standard and are appropriate to address the question. However, performance-based metrics are notorious for having inconsistent effects in ADHD, and performance may be affected by additional factors. An inclusion of an additional self-report measure of attention may have been helpful.

We agree with this point and indeed have factored this into other studies we have set up since. We have added a section into the discussion on the need for self-report but also to look at the wider impact e.g. on Quality of Life, ideally using ADHD specific measures. Clearly these scales would be more suited to studies that look at longer term impact rather than completing it once and then again ~1 hour later so we have included this as part of the limitation on the duration of the study. We hope that this is acceptable [Lines 589-595].

- Participants confirmed that they had not consumed protein-rich meals the night before; However, was intervention time fixed for all subjects? Adult ADHD is known to be associated with delayed circadian rhythmicity (see Becker, 2020; Snitselaar et al., 2017) and there is an association between circadian-related time of day and TRP (e.g., Rao et al., 1994). This is needed to be further clarified.

Testing was not fixed for all participants. Testing slots were at 10am, 1pm or 4pm so there was variety in this which adds further to the need to have ideally collected physiological data (raised above). We have added this detail on timing and noted this as a limitation [Lines 576-578]. We are grateful to the reviewer for identifying the references above, which we have also now included.

- Statistical analysis: given the null results, I wonder if a Bayesian analysis to confirm no difference would be relevant here.

We have considered this suggestion by the reviewer but have opted not to implement it for two reasons. Firstly, this is a pre-registered study and we have conducted the analysis as planned and the original plan did include an analysis suitable for the design and typically used within medical research, where arguably the most important outcome is to decide if a treatment works or not. Secondly, and we feel more importantly, we do not believe our data is suitable for Bayesian analysis because we have no prior knowledge of the data due to the lack of studies using TRP loading and the general lack of work on adults with ADHD. In the absence of priors, Bayesian analysis is inappropriate. Furthermore, without priors, or without informative priors, the confidence intervals within frequentist analysis would be equal to the credible intervals within Bayesian analysis, meaning that there is nothing to gain by this analysis. We think from the reviewer’s phrasing that this was a suggestion to considered rather than a requirement and we hope that our response indicates that we did give this consideration (and indeed confirmed our understanding with an expert statistician) and therefore that it is acceptable to not make any changes to the manuscript.

3. Results: the results are generally well described. A few points to consider:
- The Study Population section describes the exclusion / drop out reasons only vaguely – please provide accurate numbers (and %) for the different exclusion reasons. This may be important for replication purposes etc.

We had omitted this information previously because many individuals were excluded on multiple grounds. However, we appreciate this information could be important for replication and have provided a supplementary table (S1) detailing the exclusion reasons for the three groups. We have detailed the order in which criteria were reviewed, which was consistently used, and given the values for exclusions based on each item in turn. Reference to this is now made in the Study Population section [Lines 306-311, S1 Table].

I wonder if the results of any of the tests have not changed due to floor/ceiling effects, due to the young adult sample. Have the authors compared their data to published norms of these standardized tests? In other words, if they were already at peak performance to begin with, not much change is expected, especially given task learning that may happen from one administration to the other.

We investigated this by considering the studies which had previously found differences between those with ADHD and those without (and are referenced in the manuscript). As might be expected, how data is presented varies between papers making direct comparisons difficult. However, work by Barkley et al. (1996) which used the same TOVA task in young adults differed little from our findings, for example, they recorded 2.1 omission errors in their ADHD sample, and we had 2.94 and 4.59 in the medicated and unmedicated respectively. If anything, our control sample may have been performing worse than in the Barkley study but looking at the spread of the data this is unlikely to explain the differences found. Based on this, we do not believe that the TOVA data was impacted by floor or ceiling effects. For the DDT, we compared our data to that of Hurst et al. (2011) who had previously used this task with young adults, studying at university, with and without ADHD. There results showed similar average AUC for the control group (Hurst et al = 0.40, Current study 0.42 = 0.48). However, our ADHD population showed comparable AUC, whilst in the Hurst study this was lower (0.19) albeit with a large range (0.91). Comparisons were harder to make for the IGT due to differences in analysis and reporting measures. As such we compared our findings to work which examined IGT data in healthy controls (Bull et al 2015 which compared data they collected to data collected in several other studies). This indicated that our sample (all groups) may have selected from the risk decks more frequently than previous work, but the fact that prior to any intervention risky deck percentage ranged from 37% to 58% suggests that floor or ceiling effects were not impacting here. We had previously added in some discussion about the results of the tasks into the discussion in response to Reviewer 2 but we have now added in an additional section to explain this, albeit it briefly [Lines 441-451].

- The study is a double-blind RCT, but it is not clear if the blinding procedure was successful: have the authors examined blinding success with participants and/or staff? This may not be critical due to the null effects, but still important to report in a double-blind RCT.

We did not examine this with participants, but staff were not able to access the information about the blinding until data analysis point and we had no reported breaks in the blinding. We have added this information into the text [Line 525].

4. Discussion: limitations should mention the lack of more objective physiological measures and the fact that only a single consumption and immediate effects were examined, among others.

We have now added considerably to the limitations section, in line with comments from all three reviewers. This includes the points that the reviewer has raised above. The new limitations section spans Lines 548-595 which includes both points.
