# Supplementary material for: The effects of tryptophan loading on Attention Deficit Hyperactivity Disorder in adults: A remote double blind randomised controlled trial
Source: PLoS One. 2023 Nov 30;18(11):e0294911. doi: 10.1371/journal.pone.0294911 (PMC10688902; doi:10.1371/journal.pone.0294911)
Supplement: Attachment — Submitted filename: Response to Reviewers.docx [file pone.0294911.s007.docx]

**Response to Reviewers**

**Journal Requirements:**

Please review your reference list to ensure that it is complete and correct. If you have cited papers that have been retracted, please include the rationale for doing so in the manuscript text, or remove these references and replace them with relevant current references. Any changes to the reference list should be mentioned in the rebuttal letter that accompanies your revised manuscript. If you need to cite a retracted article, indicate the article’s retracted status in the References list and also include a citation and full reference for the retraction notice.

We have cross checked all the papers to be sure those in the manuscript are recorded in the list at the end (and only recorded twice). We are not aware of any of the sources we have cited being retracted. In response to the reviewers comments we have added one additional source and given that reference (and the corresponding number for the manuscript reference) in the response below.

**Reviewers' comments:**

General response: We are delighted to that all reviewers who reviewed the original manuscript and then reviewed our revisions were satisfied that all comments had been addressed. We note also that all four reviewers agreed that the manuscript:

- Was technically sound, and do the data support the conclusions.
- Included statistical analysis been performed appropriately and rigorously.
- Presented in an intelligible fashion and written in standard English.

**Specific responses:**

**Reviewer 1**

The authors have made a sincere effort to be responsive to the previous review, and also made additional clarifications to the manuscript which have improved the clarity of the presentation.

We are grateful to the reviewer for the additional review they completed and for recognising the considerable changes we made in response to the helpful feedback from all reviewers.

**Reviewer 2**

No further comments were made and therefore no response is required.

**Reviewer 3**

The authors did a great job addressing all comments made by the reviewers. I have no further comments.

We are grateful to the reviewer for the additional review and are pleased they see no further need for changes.

**Reviewer 4:**

A 3-way factorial designed randomized controlled clinical trial was conducted which aimed to evaluate the effect of acute tryptophan (TRP) loading on attention and impulsivity in adults with ADHD. No effects of TRP were observed.

Minor revisions:

We have included responses to each point below in blue. The line numbers referred to the marked-up version of the manuscript but to make things easier we have also included the amended text within each response.

1- Line 292: Define M.

We have now rephrased this sentence to ensure that we define all the abbreviations. It now reads “Descriptive data in the form of the mean (M) ± the standard deviation (SD) or count/percentage (N/%) were used to characterise the sample in terms of demographic and clinical variables with group comparisons made with ANOVAs and chi-square analyses.” [Lines 297-298]

2- State and justify the study’s target sample size with a pre-study statistical power calculation. The power calculation should include: (1) the estimated outcomes in each group; (2) the α (type I) error level; (3) the statistical power (or the β (type II) error level; (4) the target sample size, (5) the statistical testing method, and (6) for continuous outcomes, the standard deviation of the measurements.

We had already given quite detailed information on the power calculations. On lines 154 to 158 we have given the estimated effect size (based on previous work), the power (3), the statistical method (5) and the target sample size (4). The effect size was used rather than estimates of means (1) and standard deviations (6) because our calculations were based on a previous study where we had an effect size to work with. Therefore, we think the only information really missing was alpha (2) so we have now included that but rephrased the whole section to make it clearer that the information is there because we recognise it was not clear originally. Lines 154-161 now read ‘Although we initially assumed a small-to-medium effect (*f =* 0.20), based on effects across several constructs [43], we later revised this using the work by Mette et al. [48], which was closest to our paradigm, to assume a medium effect size (*f* = 0.25). Using G*Power, we calculated the required sample size with an effect size of *f* = 0.25, α (type I error level) = 0.05 and β (type II error level) for a repeated measures ANOVA, powered to detect interaction effects aligning with our hypothesis (i.e., to examine whether TRP modulation had dose dependent effects). Using these parameters, 108 participants would be required in total, which amounts to 12 per group.’

3- Cite the statistical software used for the analysis.

We have now added this information at the start of the Statistical Analysis section. The first sentence now reads ‘All analyses were completed using SPSS (IBM SPSS Statistics for Windows, Version 28.0) and performed with a two-tailed significance value of 5% and data was checked for normality using histograms, measures of skewness and kurtosis, and the Kolmogorov-Smirnov normality test.’ [Lines 293-294]

4- Age was statistically significantly different between the Controls and both ADHD groups. Comment if the statistical difference is clinically meaningful. Oftentimes when sample sizes are large, age is statistically significantly different but the difference is not clinically meaningful.

We thank the reviewer for raising this interesting point. The age gap between the control and ADHD groups was small (largest difference was 2.74 years between control and ADHD-M) but it was statistically significant and so we included it in our analysis as a covariate. We were unable to find any literature that mapped out ADHD symptomology with such a detailed time breakdown, but we believe that this difference is highly unlikely to be clinically meaningful. The ages of all participants were within a relatively narrow band and a recent study mapping out executive functions relevant to ADHD across the life span found no differences in the various measures between the ages of 23 year and 26 years which approximately aligns to our age differences [1]. We have added a sentence into the results section after we identified the difference as follows ‘Given the developmental trajectories of cognitive functions relevant to ADHD do not differ markedly in this period [78] this age difference is unlikely to be clinically meaningful’. Within the manuscript the new reference given below is reference 78 [Lines 325-327].

[1] Ferguson HJ, Brunsdon VEA, Bradford EEF. The developmental trajectories of executive function from adolescence to old age. Scientific Reports. 2021;11(1):1382. doi: 10.1038/s41598-020-80866-1.

5- Line 331: The standard statistical term for average is mean.

We have changed this to the mean to avoid any ambiguity. The sentence now reads ‘The mean adherence to medication was 90.6% (SD = 11.2)’. [Line 339]

6- Table 2 title: Summary data has been expressed “M (SD)” rather than “M +/- SD” to be consistent with the format used in the table.

Thank you for spotting this inconsistency. We have now corrected this so that the final sentence of the table title reads ‘All scores expressed as M(SD).’ Note that in making this revision, we realised that the M(SD) was also missing from one line in Table 1, for the ASRS and have now added that in as well.
